# Simultaneous Formation of a Foldamer and a Self-Replicator by Out-of-Equilibrium Dynamic Covalent Chemistry

**DOI:** 10.1021/jacs.4c09111

**Published:** 2024-11-26

**Authors:** Ankush Sood, Pradeep K. Mandal, Jim Ottelé, Juntian Wu, Marcel Eleveld, Joydev Hatai, Charalampos G. Pappas, Ivan Huc, Sijbren Otto

**Affiliations:** † Centre for Systems Chemistry, Stratingh Institute for Chemistry, Nijenborgh 3, 9747 AGGroningen,The Netherlands; ‡ Department of Pharmacy, Ludwig-Maximilians-Universität München, Butenandstraße 5-13, D-81377Munich, Germany

## Abstract

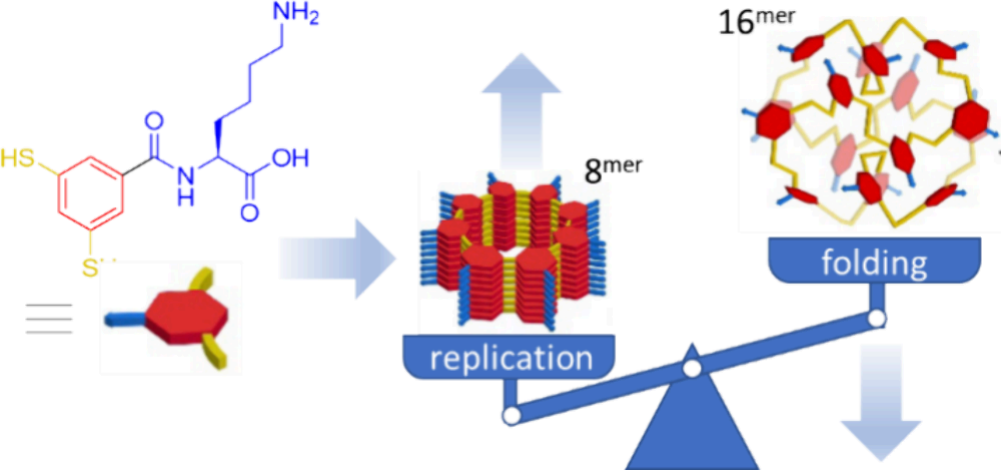

Systems chemistry has emerged as a useful paradigm to access structures and phenomena typically exhibited by living systems, including complex molecular systems such as self-replicators and foldamers. As we progress further toward the noncovalent synthesis of life-like systems, and eventually life itself, it is necessary to gain control over assembly pathways. Dissipative chemical fueling has enabled access to stable populations of (self-assembled) structures that would normally form only transiently. Here, we report a synthetic dynamic combinatorial library, made from a single structurally simple building block, from which a self-replicator and a foldamer can emerge along two distinct and competing pathways through an inter- or intramolecular assembly process, respectively. A fueled chemical reaction cycle is then set up to generate the foldamer transiently, in the presence of the self-replicator. The partitioning of the building block between the folding and self-replication pathways and the duration of the fueled reaction cycles are controlled by adjusting the amount of the chemical fuel. An out-of-equilibrium steady state involving the two assemblies could also be achieved by using a continuous stirred tank reactor with inflow and outflow of material. This work connects the domains of folding and self-replication in synthetic systems through dissipative out-of-equilibrium chemistry. It demonstrates that foldamers and self-replicators, formed from the same building block, can stably coexist if the system is continuously supplied with energy, while at equilibrium, the Gibbs phase rule prohibits such coexistence.

## Introduction

Macromolecules that fold or replicate occupy central roles in the biochemical processes that sustain life.^1^ In nature, the coexistence and functional integration of folded and replicating macromolecules are implemented with two different compound classes, i.e., oligomers of amino acids and oligonucleotides, respectively. These compound classes are connected through the complex and highly evolved processes of transcription and translation. How such a complex interplay between folded molecules and replicating ones can have emerged in the early evolution of life remains an open question.

Individually, foldamers and replicators can be formed from relatively simple starting materials. While several early reports on artificial self-replicating^2−8^ and folding structures^9−14^ relied on elaborate synthetic planning, lately, a systems chemistry approach revealed that such molecules can also emerge spontaneously.^15−19^ This approach is exemplified through dynamic combinatorial libraries (DCLs) which are collections of molecules formed by connecting building blocks through dynamic covalent bonds.^20^ An exchange between building blocks renders these systems dynamic and endows them with properties like error correction and responsiveness. Interaction of library members with an externally added template or with other library members can alter the composition of DCLs as the overall Gibbs energy of the system is minimized.^21,22^

We have previously shown the spontaneous generation of self-replicating^17,19,23,24^ or folding structures^18,25^ made upon oligomerization of synthetic building blocks. The building blocks from which both self-replicators and foldamers formed feature a 1,3-dimercaptobenzoic acid core that is functionalized at the carboxylic acid group with a fragment (e.g., a peptide, a peptide-nucleic acid conjugate, or a short oligoethylene glycol chain) that influences the assembly behavior of the disulfide macrocycles formed upon oxidation of the thiols. The use of fragments consisting of a pentapeptide with alternating hydrophobic and hydrophilic amino acids favors supramolecular polymerization^8,26−28^ of the resulting macrocycles through the formation of β-sheets, in addition to π-stacking of the aromatic cores. A nucleation–elongation mechanism, coupled with fiber breakage, enables exponential self-replication of the assembling macrocycle.^29^ The strength of the β-sheet interactions diminishes with decreasing peptide length. When using dipeptides, the folding of macrocycles driven by hydrophobic and π-stacking interactions allows for an alternative stabilization mechanism that shields the hydrophobic aromatic core from the aqueous environment.^25^

The use of DCLs to obtain a single assembly of a specific nature is now relatively well established, exploiting the fact that the assembly that yields the lowest Gibbs energy for the system is likely to form in excess over any competing structures. However, competing assemblies that correspond to less favorable Gibbs energies may also be of interest. Yet, for assemblies made from multiple identical building blocks held together by dynamic covalent bonds, the Gibbs phase rule predicts that, at equilibrium, only a single phase corresponding to the specific assembly will dominate, and stable coexistence between two or more phases is prohibited under these conditions (if we disregard the special situation of residing exactly at a phase boundary).^30−32^ This raises the question of whether assemblies, differing from that corresponding to the thermodynamic minimum, can be accessed by maintaining DCLs away from equilibrium. It also raises the question of whether different assemblies can stably coexist under out-of-equilibrium conditions, even when they are made from the same building block. We are not aware of any such systems in the literature.

Recently, we made two discoveries that showed that pathways leading to folded molecules or to self-replicating molecules can exist simultaneously in a DCL. First, in a two-building-block DCL, we discovered a self-sorted mixture with a self-replicator incorporating both the building blocks and a foldamer incorporating only one of the building blocks.^33^ This outcome was dictated by stoichiometric constraints of the system: the foldamer only formed from leftover building blocks that could not be incorporated into the replicator. Second, we found that a building block that formed a foldamer with 23 identical subunits in aqueous buffer, produced a self-replicating hexamer when 1.0 M of sodium bromide salt was used.^34^ In the absence of salt, the system remained kinetically trapped in a folded state, but partial reduction, to reinvigorate disulfide exchange, altered the distribution irreversibly and completely to a self-replicating hexamer macrocycle. In both cases, control over ratios of folded and self-replicating macrocycles and their lifetimes remained elusive. Furthermore, stable coexistence of two assemblies made from the same building block was not possible under the conditions used.

Motivated by these discoveries, we set out to construct a system where both folding and self-replicating pathways are accessible simultaneously from a single building block, and control over partitioning of the building block along two pathways is possible. Building on our earlier experiments on mass-transport flow to escape thermodynamic control,^35,36^ we reasoned that such out-of-equilibrium conditions should allow stable coexistence of the two assemblies.

Dissipative chemistry has emerged in the last two decades as a powerful approach to obtain systems and phenomena that would not be accessible at equilibrium, including transient assemblies,^37−39^ pH gradients,^40^ oscillations,^41^ and unidirectional motion around a chemical bond.^42^ These systems rely on an input of energy in the form of light or a supply of high-energy molecules (fuels) to carry out transformations that generate products or phenomena transiently. While numerous examples of dissipative formation of an assembly from nonassembling precursors have been reported recently,^43,44^ dissipative interconversion between different assembly types is still, for as far as we are aware, without precedent.

We now report a system, made from a single building block, that features two competing assembly paths leading to folding or self-replication. This complex behavior emerges from the simplest molecule that has yet to be reported to autonomously fold or replicate. Tuning the experimental conditions allows for the complete conversion of the building block into either a self-replicator or a foldamer. When using a chemically fueled reaction cycle, the foldamer can be generated transiently in the presence of the self-replicator and the ratio of the two species and the duration of the fueled reaction cycle can be tuned by adjusting the amount of chemical fuel. Using a mass-transport flow setup, an out-of-equilibrium steady state with a product distribution that favors the thermodynamically less stable foldamer is achieved. Our results show that stable coexistence of folded and self-replicating structures, made from the same building block, is possible if the system is maintained out of equilibrium through a continuous supply of energy.

## Results and Discussion

### Building Block Design and Screening

When DCLs were targeted with the propensity to exhibit competing folding and self-replication pathways, building blocks with a single amino acid appended to 1,3-dimercaptobenzoic acid were attractive starting points for two reasons (besides relative ease of synthesis). First, shorter peptides are sterically easier to incorporate around a folded aromatic core while probably still large enough to allow the peptide fragment to shield the hydrophobic core from the aqueous environment. Our designs targeting new foldamers have thus far focused on dipeptides with at least one amino acid carrying an aromatic side chain, which can promote folding due to π-stacking between the core and the peptide.^18^ Second, while assembly-driven self-replicating macrocycles are predominantly observed for longer pentapeptides, in a rare example, a phenylalanine appended core could form a self-replicating hexamer macrocycle, but only when assisted by amine templates.^45^ Note that the 1,3-dimercaptobenzene core appears to be privileged, since analogs with a 1,4 substitution pattern^46^ or analogs with −CH_2_SH groups^47^ thus far failed to produce foldamers or replicators.

**Figure 1:**
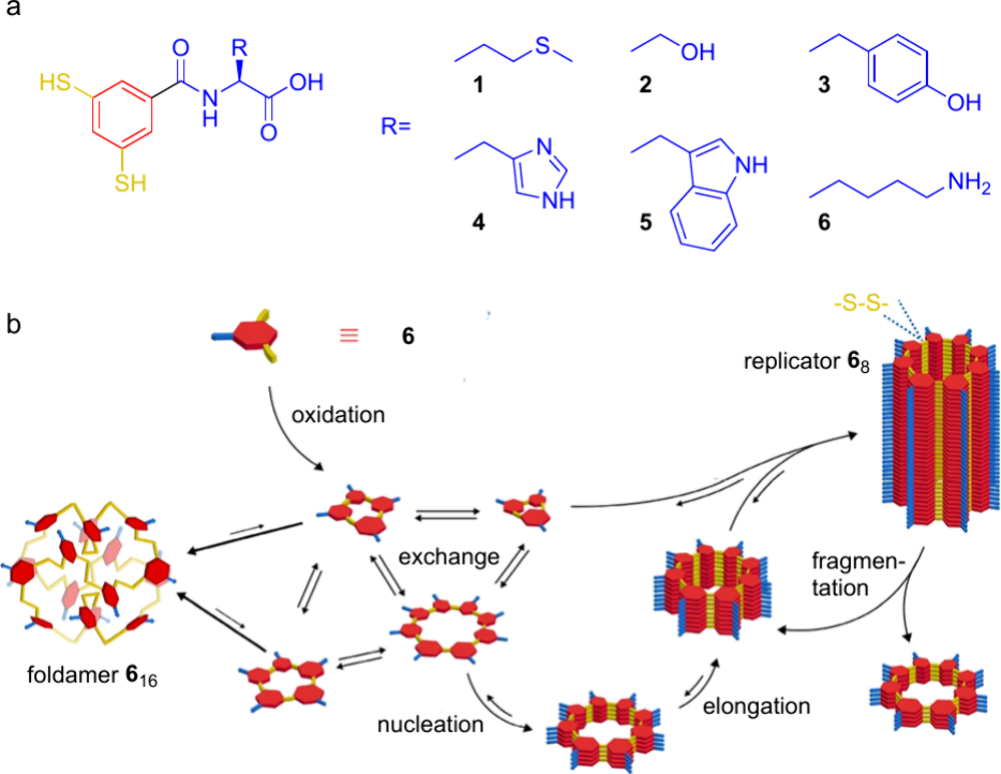
(a) Building blocks used to prepare dynamic combinatorial libraries targeting competing self-replication and folding pathways. 1,3-Dimercaptobenzoic acid is appended to amino acids with different propensities to stabilize supramolecular fibers and foldamers. (b) Assembly pathways in the DCL made from **6**. Oxidation of building block **6** generates a collection of interconverting macrocycles. Intra- or intermolecular assembly, giving rise to foldamers or fibers, respectively, stabilizes the corresponding macrocycles shifting the library composition toward these specific species.

For screening, we ruled out amino acids carrying aliphatic side chains without a heteroatom. This was based on earlier observations that amino-acid side chains capable of hydrogen bonding and ionic interactions tend to stabilize foldamers^25^ and further that the use of spermine as a template could induce fiber formation in a DCL made from a building block containing single phenylalanine.^45^ In the latter case, ionic interactions between the protonated amines of spermine and the carboxylate group of the building block can most likely stabilize fibers in a manner similar to the way salt bridge interactions stabilize both foldamers^25^ and fibers.^48^ The structures of the building blocks explored in this study are shown in Figure 1a.

After 3 weeks oxidation in air, DCLs made from methionine (**1**) and serine (**2**) containing building blocks, analyzed by ultraperformance liquid chromatography–mass spectrometry (UPLC-MS; Figures S2 and S4), showed predominantly entropically favored trimer and tetramer macrocycles that did not appear to form any ordered aggregates (analyzed by negative staining transmission electron microscopy (TEM)) under the conditions of our experiment (0.45 mM in building block, 50 mM in borate buffer, pH = 8.0, 30 °C, constant stirring). We then investigated building block **3** that contains tyrosine, which, unlike phenylalanine, can participate in hydrogen bonding.^49^ A nonamer macrocycle **3**_9_ (40%) was observed as the dominant species, besides appreciable amounts of trimer and tetramer macrocycles (Figure S6). The formation of such a large macrocycle typically hints at stabilization from some form of assembly since its formation is entropically unfavorable compared to smaller macrocycles. We speculated that **3**_9_ could be folded in accordance with our earlier observations^25,50^ in which a macrocycle of the same size was found to fold. Further, we did not observe any (fibrous) aggregates for this sample.

We then proceeded with histidine-containing building block **4**, which, besides hydrogen bonding, can also form salt bridges^51^ with carboxylates. A macrocyclic hexadecamer **4**_16_ (25%) was observed along with cyclic trimers and tetramers (Figure S8). Such a selectivity in size, again, hinted that **4**_16_ could be folded. As a control, we also studied a building block containing tryptophan (**5**), since it is aromatic but not ionizable. We did not see appreciable amounts of macrocycles larger than trimers and tetramers (Figure S10).

Even as DCLs of building blocks **3** and **4** produced large macrocycles whose sizes corresponded to foldamers discovered earlier, they did not show any evidence of a competing self-replication pathway. We reasoned that this could be either because the interactions between macrocycles are insufficient to allow their nucleation into stacks, or the libraries are kinetically trapped in a folded state. The probability of the latter would increase with decreasing propensity of foldamer to (partially) unfold in response to environmental stress (see below). To test the latter hypothesis, we substituted histidine with lysine as this can potentially reduce the stability of the foldamer by reducing the propensity for π-stacking while retaining the ability to engage in ionic interactions. Indeed, competing folding and self-replication pathways were observed in a DCL fabricated from **6**.

### Competing Self-Replication and Folding Pathways from a Single Building Block

**Figure 2:**
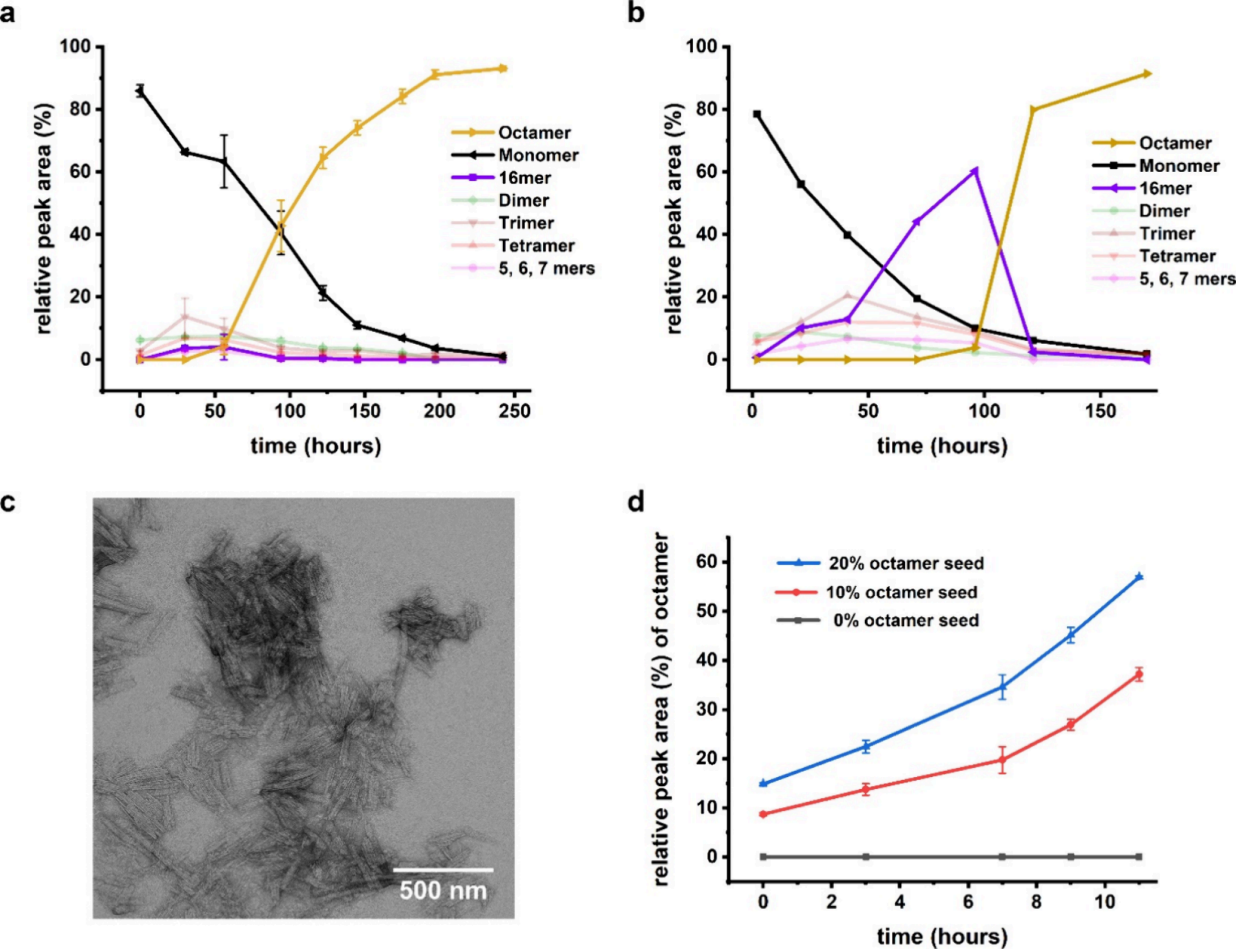
Formation of a self-replicating octamer macrocycle **6**_8_ from a dynamic combinatorial library (DCL) made from building block **6** (0.45 mM in building block, 50 mM in borate buffer, pH = 8.0, 30 °C, constant stirring). (a) Change in the composition of DCL of **6** monitored by UPLC (absorbance at 254 nm): **6**_8_ grows following a lag phase and then becomes the dominant species. Presence of a hexadecamer macrocycle **6**_16_ hints at a possible folding pathway; (b) late nucleation of **6**_8_ allows for transient enrichment of **6**_16_; (c) negative staining TEM images of **6**_8_ samples show the presence of laterally associated fibers. (d) Addition of preformed **6**_8_ samples (20% and 10% v/v) to a partially oxidized solution (50% thiol oxidation) of **6** leads to an immediate increase in **6**_8_ concentrations. The increase is faster when a larger amount of preformed **6**_8_ is added, indicative of self-replication by **6**_8_. Lines are drawn to guide the eye.

Kinetic studies of a DCL of **6** with UPLC-MS revealed rich assembly behavior (Figure 2a–d). The concentration of an octamer macrocycle **6**_8_ increased following a lag phase that lasted between one and 2 days before rapid growth over the next 150 h made it the dominant species in 10 days. The sigmoidal growth (Figure 2a,b) points toward a nucleation-elongation mechanism, even though the growth rate eventually becomes limited by the slow oxidation of the building block. The presence of a small amount of hexadecamer **6**_16_ in the early stages of the library did not escape our attention. It is noteworthy that we failed to detect any macrocycle size between octamer and hexadecamer by UPLC-MS.

While the lag phase for **6**_8_ typically lasted 2 days, in rare instances, it was much longer. This could be due to the stochastic nature of nucleation processes.^52^ The longer lag phase for the formation of **6**_8_ allowed **6**_16_ to transiently dominate library composition (Figure 2b) before getting depleted upon growth of **6**_8_. Encouraged by these observations, we decided to further investigate the different assembly pathways in the DCLs of **6**.

We first carried out structural and mechanistic investigations on **6**_8_ that could be readily isolated in good purity. TEM analysis for **6**_8_ samples revealed laterally associated fibers that were roughly 100 to 300 nm in length (Figure 2c). A seeding experiment was performed to study the mechanism of assembly (Figure 2d): different amounts of preformed **6**_8_ fibers were added to a 50% preoxidized library of **6** in which **6**_8_ had not yet nucleated. Only the samples with seed showed an immediate increase in the level of **6**_8_, suggesting a nucleation-elongation mechanism. Further, the rate of growth of **6**_8_ increased with increasing amounts of **6**_8_ seeds, confirming that it is a self-replicating macrocycle.

**Figure 3:**
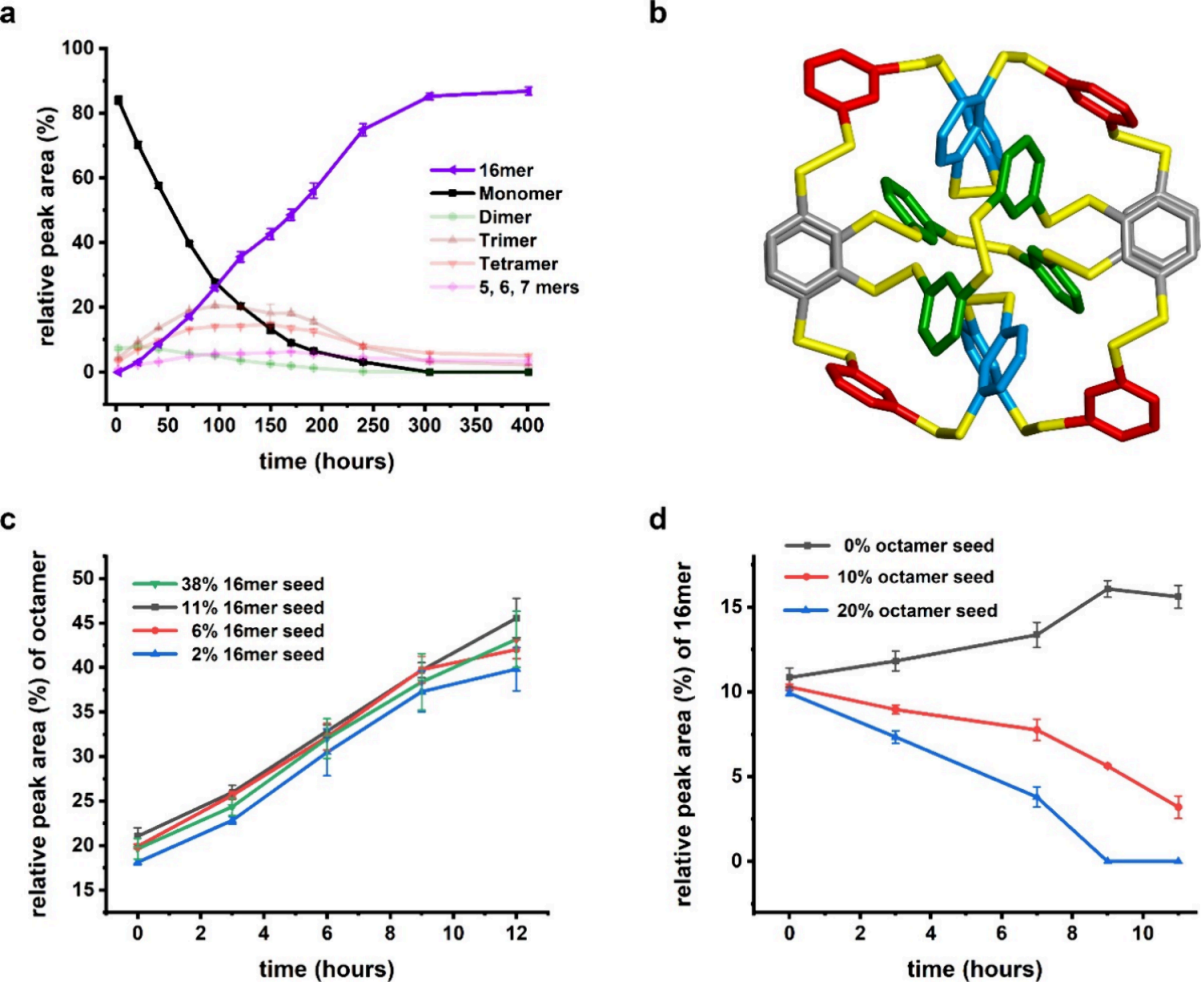
Macrocycle **6**_16_ is a foldamer that can form in a DCL made of **6** through an alternative pathway than that leading to self-replicator **6**_8_. (a) Change in composition of a DCL made from **6** monitored by UPLC (absorbance at 254 nm) in the absence of mechanical agitation. (b) X-ray diffraction analysis confirms that **6**_16_ is a foldamer. Shown here is the folded structure of the core of disulfide-linked aromatic rings of the d enantiomer of **6**_16_ viewed down its crystallographic *C*_2_ axis. (c) Initial rate of formation of **6**_8_ is unaffected by the amounts of **6**_16_ added at *t* = 0, suggesting that **6**_16_ is not promoting the formation of itself or of **6**_8_; (d) Addition of **6**_8_ at *t* = 0 leads to depletion of **6**_16_. Lines are drawn to guide the eye.

Next, we investigated the structure and formation mechanism of **6**_16_. However, isolating it was a challenge due to its transient nature. We reasoned that modifying the preparation method might allow DCLs of **6** to become kinetically trapped into **6**_16_. Self-replication in this system progresses exponentially if fibers break to yield more fiber ends, which are the sites of fiber growth.^29^ Self-replication can happen without mechanical agitation but is expected to be much slower under such conditions.^17,53,54^ Indeed, we were able to selectively form **6**_8_ or **6**_16_, depending on whether or not samples were agitated (Figures 2a and 3a). Unlike **6**_8_, **6**_16_ showed nonsigmoidal kinetics suggesting the absence of an autocatalytic growth mechanism. Furthermore, we did not observe fibers in negative-staining TEM for **6**_16_ samples or accelerated growth upon seeding. Samples of **6**_8_ and **6**_16_ showed distinct CD spectra (Figure S35), and, while samples of **6**_8_ incubated with thioflavin T dye showed enhanced fluorescence emission, **6**_16_ samples did not show such enhancement, suggesting the absence of long-range ordered aggregates (Figure S36). MALDI-TOF mass spectrometry of **6**_16_ ruled out the possibility of **6**_16_ being a quaternary or interlocked structure of two or more macrocycles (Figure S37) as it fragmented in a continuum of progressively shorter fragments.

We isolated **6**_16_ with automated flash chromatography and attempted to crystallize it. As our initial attempts failed to produce a diffraction quality crystal, we proceeded with isolating also the d enantiomer of **6**_16_, enabling the crystallization of racemic **6**_16_.^55^ Much to our surprise, only the d enantiomer crystallized from the racemic mixture. Figure 3b shows the hydrophobic aromatic core of **6**_16_ in a folded conformation with a crystallographic 2-fold symmetry obtained upon refinement of the X-ray diffraction data. The fold is stabilized by π-stacking between two pairs of phenyl rings seven residues apart (Figure S40). Peripheral lysine residues, which are disordered, remain exposed to an aqueous environment and partially shield the aromatic core (Figure S39d). Obvious favorable interactions between the Lys residues or between Lys residues and the hydrophobic core are far fewer than in related foldamers with dipeptide appendages,^25,50^ making it remarkable that this specific ring size actually emerges. The hydrodynamic radius (1.5 nm) of **6**_16_, calculated from diffusion-ordered NMR experiments (Figure S43), corresponds with the radius obtained from X-ray diffraction data (1.25 nm), suggesting that **6**_16_ is folded in solution as well.

The fold of the hydrophobic core of **6**_16_ displays conformational polymorphism with respect to two superimposable hexadecamer cores reported earlier for DCLs made from dithiol building blocks featuring either dipeptide Phe(4-guanidinium)-Lys,^25^ or Phe(4-CO_2_H)-Lys.^50^ Specifically, the *P* or *M* chirality of some disulfide bonds is different in the two types of structures (Figure S42). The relative stabilities of the two types of hexadecamers are also markedly different, possibly due to the absence of aromatic amino acids in **6**_16_. Variable temperature CD analysis shows that **6**_16_ begins to unfold at a much lower temperature–around 45 °C (Figure S44) compared to 75 °C for the dipeptide-containing hexadecamer. UPLC analysis at varying sample temperatures suggests that the loss in CD signal is due to partial unfolding of **6**_16_ (Figure S45) and not due to changes in covalent bond/ring size distribution.

To establish whether folding and self-replication in the DCL of **6** occur through two distinct pathways, we studied the kinetics of **6**_8_ growth as a function of initial **6**_16_ concentrations and vice versa. Increasing initial **6**_16_ concentrations at a fixed initial amount of **6**_8_ did not substantially alter the rate of growth of **6**_8_ (Figure 3c), suggesting that **6**_16_ is not an intermediate in the formation of **6**_8_ or otherwise promoting its formation. When different initial amounts of **6**_8_ were added to samples at the same initial **6**_16_ concentrations, depletion of **6**_16_ was fastest when larger amounts of **6**_8_ were present (Figure 3d). Taken together, these observations establish the presence of two competing pathways with foldamer **6**_16_ as the metastable state. Figure 1b shows the assembly pathways present in DCL of **6**.

### Fueling Allows Control over Folding and Self-Replication from a Single Building Block

The data in Figure 3c,d suggest that **6**_16_ is metastable relative to **6**_8_. Once self-replication of **6**_8_ begins, the product distribution changes completely and irreversibly to give **6**_8_. This behavior prevents obtaining a state where foldamer and self-replicator stably coexist. As, upon approach to equilibrium, the foldamer concentration diminishes when the replicator is present, energy input is necessary to (re)generate the foldamer and a continuous energy input would be required to obtain it in a steady-state concentration alongside the replicator.

We first probed whether it would be possible to form foldamer **6**_16_ from replicator **6**_8_ upon consecutive addition of reducing agent tris(2-carboxyethyl)phosphine hydrochloride (TCEP·HCl) and oxidizing agent sodium perborate (NaBO_3_) as redox fuels. The former reduces disulfides to thiols and produces the corresponding phosphine oxide as waste, while the latter oxidizes thiols to disulfides, generating sodium borate as waste. Reduction and oxidation therefore are not each other’s microscopic reverse, which allows the system to be pushed away from equilibrium. We have previously used similar redox fueling in DCLs of disulfides to convert a thermodynamically favored structurally simple self-replicator into a thermodynamically less stable more complex replicator.^35^

**Scheme 1:**
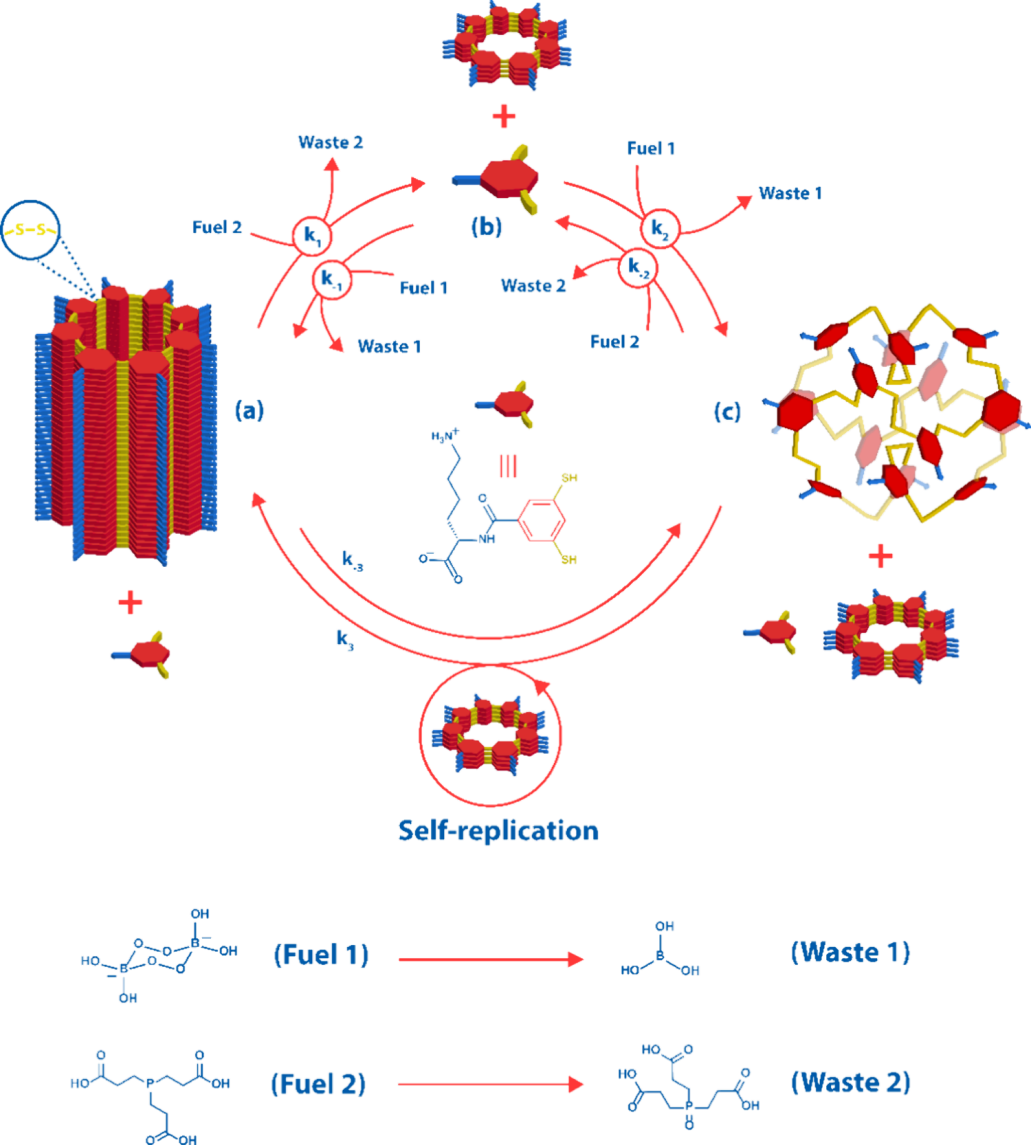
Fueled Reaction Cycle that Generates Foldamer Transiently from Self-Replicator Self-replicating **6**_8_ (a) is converted to monomer **6** (b) by reducing agent TCEP (fuel 2). Subsequent oxidation of **6** by oxidizing agent sodium perborate (fuel 1) generates folded **6**_16_ (c) rapidly and selectively. Subsequent self-replication of **6**_8_ consumes **6**_16_ and regenerates **6**_8_, completing the cycle. For clarity, for the reactions coupled to fuel-to-waste conversion, we did not show the reactions that are their microscopic reverse, as these reactions are highly endergonic.

We compared the reactivity of fibrous **6**_8_ and folded **6**_16_ toward reduction by reacting an equimolar solution of **6**_8_ and **6**_16_ with TECP. We observed a selective reduction of **6**_16_ in the presence of **6**_8_ (Figure S47). Previous work on the reduction of replicators indicated that TCEP reacts predominantly with disulfides exposed at fiber ends, while those buried in the fiber interior were not readily accessible.^56^ It appears that (at least part of) the disulfides in foldamer **6**_16_ are more accessible than those in the fibers of **6**_8_. The perborate-mediated oxidation of the building block produced **6**_16_ with remarkable selectivity, without significantly increasing **6**_8_. Together, these differences in reactivities allowed us to design the fueled reaction cycle shown in Scheme 1.

**Figure 4:**
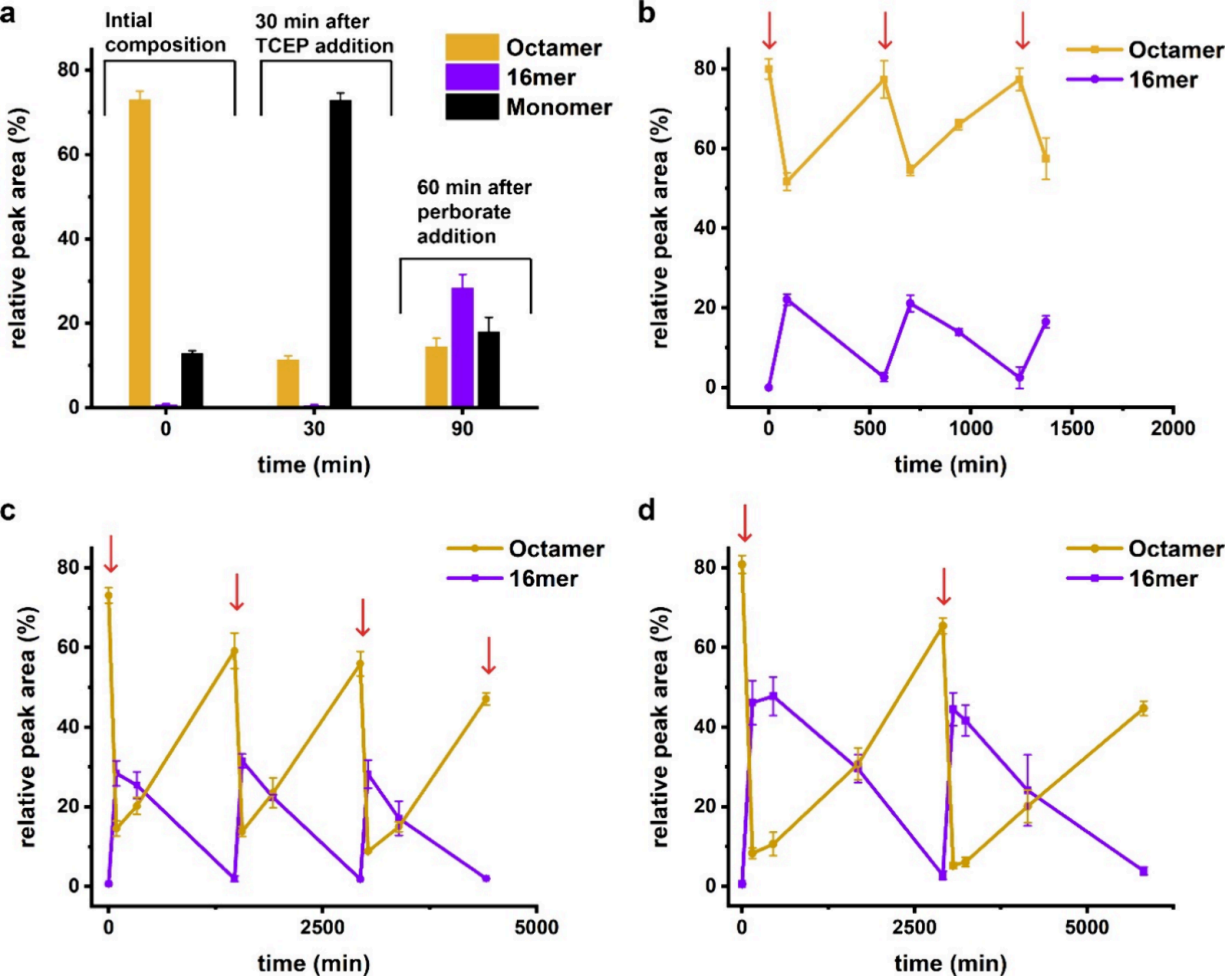
Fueled reaction cycles allow control over partitioning of the building block into folded **6**_16_ and self-replicating **6**_8_. (a) Sequential addition of TCEP followed by sodium perborate (0.6 equiv each) leads to populating folded **6**_16_. The relative amounts of **6**_16_ and **6**_8_ and the duration of the reaction cycles can be altered by changing the amounts of the chemical fuels, for example, by adding (b) 0.5, (c) 0.6, and (d) 0.7 equiv of both reductant and oxidant. Red arrows show the times at which TCEP was added. Perborate was added 30 min after the addition of TCEP. Sample compositions were analyzed by UPLC 60 min after the addition of perborate. Lines are drawn to guide the eye.

We started with a DCL of **6**, which had equilibrated to fibrous **6**_8_. Upon addition of 0.6 equiv of TCEP (relative to the total concentration of **6**), a substantial fraction of **6**_8_ was reduced, liberating building block **6** (Figure 4a). Upon subsequent addition of 0.6 equiv of perborate, a substantial fraction of **6** was converted to foldamer **6**_16_. The foldamer was then slowly converted back to the replicator (Figure 4c). The reaction cycle was repeated several times, with some fatigue in the regeneration of the replicator (possibly due to the accumulation of waste). The amounts of the two redox fuels that were added significantly affected the ratio between foldamer and self-replicator obtained immediately after the addition of fuels, from approximately 0.20 upon adding 0.5 equiv of fuel to 2.8 when 0.7 equiv of fuel was used (see Figure 4b,d, respectively). The depletion of the foldamer is coupled to the autocatalytic self-replication of **6**_8_. Therefore, the foldamer depletes more slowly when smaller amounts of **6**_8_ are present (Figure 4b–d).

This system does not exhibit an obvious kinetic asymmetry. Even though the perborate-mediated oxidation of the monomer to yield the foldamer is faster than the corresponding conversion of the monomer into replicator (*k*_2_ > *k*_–1_), the TCEP mediated reduction of the foldamer is faster than the corresponding conversion of the replicator (*k*_–2_ > *k*_1_). This allows for small amounts of the replicator to persist throughout the cycle, which is beneficial, as the replicator promotes its own formation from the foldamer.

**Figure 5:**
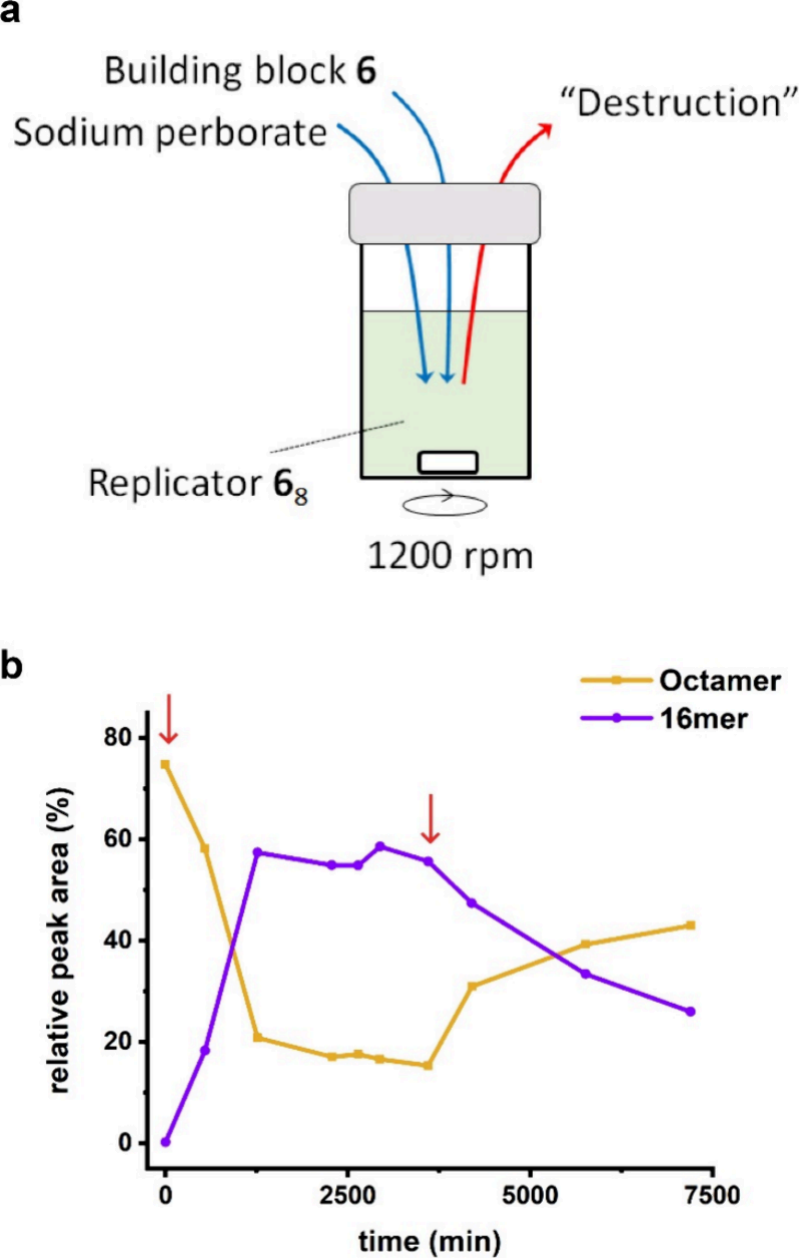
Out-of-equilibrium steady state showing stable coexistence of self-replicator **6**_8_ and foldamer **6**_16_. (a) Schematic description of the setup. Building block **6** and oxidizing agent sodium perborate are flown into the continuous stirred tank reactor containing **6**_8_. Outflow by drawing out solution removes all species indiscriminately. (b) Change in concentrations of **6**_8_ and **6**_16_ as measured by UPLC. Start and stop of flow are shown by red arrows. Total flow rate was maintained at 50 μL h^–1^ (turnover time of 600 min). Lines are drawn to guide the eye.

While the addition of redox fuels allows for dynamic reaction cycles, it was not practical to achieve stable coexistence of foldamer and replicator using this approach, as continuous addition of oxidant and reductant would act predominantly on the foldamer, as this is the compound that is fastest to be decomposed by reduction and formed by oxidation. However, a steady state of coexistence could be realized using a continuous stirred tank reactor (CSTR) in which building block **6** and perborate were continuously supplied, while part of the reaction mixture was continuously removed (see Figure 5a; operational details of the setup are described in the SI, Section 1.6).

We placed a DCL of **6** (0.45 mM) that had equilibrated to **6**_8_ inside the CSTR. Two syringe pumps were used to continuously supply building block **6** (0.90 mM) and sodium perborate (0.90 mM), separately. A third syringe was used to remove material from the CSTR. The destruction step (outflow) is indiscriminate, unlike reduction by TCEP. As sodium perborate-mediated oxidation of **6** produces **6**_16_ rapidly, along with other smaller macrocycles, the formation of **6**_16_ competes with the self-replication of **6**_8_. The latter also consumes **6**_16_. At appropriate flow rates, the balancing of the rates of formation, interconversion, and outflow of the various macrocycles should cause the concentrations of the various species to converge on an out-of-equilibrium steady state in which replicator and foldamer stably coexist. This was realized at a total flow rate of 50 μL h^–1^ (inflow rate was maintained at 25 μL h^–1^ for building blocks and oxidizing agent and outflow rate was kept at 50 μL h^–1^ for conservation of mass; turnover time was 600 min). The concentration of **6**_8_ decreased upon starting the flow but stabilized after about two turnover times (Figure 5b). By this time, thermodynamically less stable **6**_16_ was the dominant species. Once the flow was stopped after six turnover times, the concentration of **6**_8_ increased steadily but did not reach the initial concentration on the time scale of the experiment. This slow re-equilibration was likely due to the depletion of thiols necessary for the conversion of **6**_16_ to **6**_8_ by residual sodium perborate.

## Conclusions

Replicating and folding macromolecules play central roles in living systems. Complex transcription and translation machinery mediates the functional integration of these compound classes in current biochemistry. It is unclear how replicators and foldamers could have become coupled in early forms of life prior to the advent of transcription and translation. Here, we have probed a simple scenario in which coupling takes place by sharing a common building block that can give rise to a foldamer and a replicator. We identified a building block from a small screening campaign that was able to form a replicator as well as a foldamer. However, at equilibrium (and away from phase boundaries), the Gibbs phase rule precludes the coexistence of two such oligomers when these are both constituted from the same building blocks. Indeed, experiments showed that the foldamer would give way to the replicator when a mixture of the two was allowed to equilibrate. However, by operating the system away from equilibrium, it was possible to reverse this behavior. The sequential addition of reductant and oxidant to the replicator induced a reaction cycle in which foldamer was formed transiently but eventually was converted back to the replicator. Operating the systems in a flow reactor, where oxidant and building blocks were continuously supplied, while part of the reaction mixture was flown out, allowed a steady state to be attained in which foldamer and replicators stably coexist. Such systems might be a primitive and simple way of coupling genotype (self-replicators) with the phenotype (folded molecules). Once the phenotype would show functions (for example, catalysis) that would benefit the replicator, such coupling could be selected for in the course of evolution.

## Supporting Information Available

General procedures, UPLC-MS, (variable temperature) circular dichroism, thioflavin T fluorescence, MALDI fragmentation, X-ray crystallography, DOSY-NMR data, and reduction and oxidation experiments (PDF)
