# Supplementary material for: Simultaneous Formation of a Foldamer and a Self-Replicator by Out-of-Equilibrium Dynamic Covalent Chemistry
Source: J Am Chem Soc. 2024 Nov 26;146(49):33386–94. doi: 10.1021/jacs.4c09111 (PMC11638962; doi:10.1021/jacs.4c09111)
Supplement: Supplementary file 1 — ja4c09111_si_001.pdf [file ja4c09111_si_001.pdf]

Supplementary Materials for

Simultaneous Formation of a Foldamer and a Self-Replicator by Out-of-Equilibrium Dynamic Covalent Chemistry

Ankush Sood,† Pradeep K. Mandal,‡ Jim Ottelé,† Juntian Wu,† Marcel Eleveld,† Joydev Hatai,† Charalampos G. Pappas,† Ivan Huc,*‡ and Sijbren Otto*†

†Centre for Systems Chemistry, Stratingh Institute for Chemistry, Nijenborgh 3, 9747 AG Groningen, The Netherlands
‡Department of Pharmacy, Ludwig-Maximilians-Universität München, Butenandstraße 5-13, D-81377 Munich, Germany

Contents

1. General procedures
   1.1 UPLC methods
   1.2 UPLC-MS methods
   1.3 Preparation of dynamic combinatorial libraries (DCL)
   1.4 Seeding experiment of $\mathbf{6}_8$ samples
   1.5 Reaction cycles involving chemical fuels
   1.6 Continuous flow experiment
   1.7 Negative staining Transmission Electron Microscopy (TEM)
2. UPLC data
3. Peak identification with UPLC-MS
4. Circular dichroism (CD) spectroscopy for $\mathbf{6}_8$ and $\mathbf{6}_{16}$
5. Thioflavin T (ThT) fluorescence assay on $\mathbf{6}_8$ and $\mathbf{6}_{16}$
6. MALDI fragmentation pattern of $\mathbf{6}_{16}$
7. X-ray crystallography of $\mathbf{6}_{16}$
8. DOSY NMR spectra of $\mathbf{6}_{16}$
9. Variable temperature response – CD and UPLC - of $\mathbf{6}_{16}$ in solution
10. TCEP reduction of a mixture of $\mathbf{6}_8$ and $\mathbf{6}_{16}$
11. References

1. General procedures

Boric anhydride (99.98% trace metal basis) and sodium hydroxide (BioXtra, ≥98%), used for preparation of borate buffer, sodium perborate tetrahydrate (purum, 96%), used for oxidation of the thiol building block, tris(2-carboxyethyl)phosphine hydrochloride (TCEP.HCl) (BioUltra, ≥98% (NMR)), used for reduction of disulfide libraries and thioflavin-T, used for fluorescence assays, were purchased from Sigma-Aldrich.

Acetonitrile (ULC-MS), trifluoroacetic acid (ULC-MS) and water (ULC-MS), used for UPLC and UPLC/MS measurements, were purchased from Biosolve BV.

Building blocks **1-5** were purchased from GenScript Biotech (Netherlands) B.V. with 95-98% purity. Building block **6** was purchased from Cambridge Peptides Ltd. (Birmingham, UK) with 98% purity. Building blocks were used without further purification.

1.1 UPLC methods

Waters Acquity H-class with a PDA detector and Aeris Peptide column (reversed-phase, XB-C18, 1.7 μm, 100 Å, 150 mm x 2.1 mm) from Phenomenex B. V. were used for UPLC analyses. Empower and Origin were used for data processing and analysis. Solutions of 0.1 V/V % of trifluoroacetic acid (ULC-MS) with acetonitrile (ULC-MS) and with water (ULC-MS) were used as mobile phases. UV absorbances were monitored at 254 nm. Column temperature was 35°C. Sample holder temperature was 25°C.

UPLC samples were prepared in a 250 μL UPLC insert with mandrel interior and polymer feet (5.7mm x 29mm) by diluting 15 μL of the library sample with 45 μL of water (ULC-MS) to give 0.11 mM sample in building block concentration.

Method A was used for analysis of DCLs made from building blocks **2**, **3** and **5**. Method B was used for analysis of DCLs made from building blocks **1**, **4** and **6**. The gradients were linear.

Method A

time (min)	flow rate (mL min^{-1})	%A	%B
0	0.3	90	10
1.0	0.3	90	10
1.3	0.3	75	25
3.0	0.3	72	28
11.0	0.3	10	90
11.5	0.3	5	95
12.0	0.3	5	95
12.5	0.3	90	10
17.0	0.3	90	10

Method B

time (min)	flow rate (mL min^{-1})	%A	%B
0	0.3	90	10
1.0	0.3	85	15
5.0	0.3	65	35
5.5	0.3	30	70
13.0	0.3	5	95
13.5	0.3	5	95
14.0	0.3	90	10
17.0	0.3	90	10

1.2 UPLC-MS method

A Waters Acquity UPLC H-class system coupled to a Waters Xevo-G2 TOF was used. The mass spectrometer was operated in the positive electrospray ionization mode. Capillary, sampling cone, and extraction cone voltages were kept at 2.5 kV, 30 V, and 4 V, respectively. Source and desolvation temperatures were set at 150°C. Nitrogen was used as both cone (5 L h^{-1}) and desolvation gas (500 L h^{-1}). MassLynx was used for data processing and analysis.

1.3 Preparation of dynamic combinatorial libraries (DCLs)

Borate buffer (50 mM, pH = 8.2) was prepared by weighing 3.48 g of boric anhydride and adding 900 mL of water (ULC-MS). The pH was then adjusted to 8.2 using 1.0 M sodium hydroxide solution prepared by dissolving 4.0 g of sodium hydroxide in 100 mL of water (ULC-MS). The buffer solution was then made to 1 L with additional water (ULC-MS).

Building blocks **1-6** were weighed (1 mg) separately and borate buffer (50 mM, pH = 8.2) was added to obtain 0.45 mM of the building block solutions. Since building blocks **4** and **6** exist as trifluoroacetic acid salts, care was taken during molarity calculations. These solutions (500 µL) were then taken in 2 mL UPLC vials (11.6 mm x 32 mm) with Teflon-coated screw cap. Teflon coated magnetic cylindrical stirrer bars (length 7 mm and diameter 2 mm) obtained from VWR were added to each vial. The solutions were stirred at 1200 rpm using IKA RCT basic hot plate stirrer in presence of oxygen from the air. The temperature was maintained at 30°C by placing the vial inside a custom milled aluminium block in Thermo Fischer compact dry bath kept over the IKA stirrer.

1.4 Seeding experiment of $\mathbf{6}_8$

A solution of **6** (0.45 mM) was prepared by weighing 1 mg of **6** and adding 5200 µL of borate buffer. The solution was oxidised by adding 0.5 equivalent (29.25 µL) of a solution of sodium perborate (40 mM) in water, prepared by adding 12.3 mg of sodium perborate tetrahydrate to 2 mL water. After one hour from addition of sodium perborate solution, three 1500 µL solutions were taken from the partially oxidised library solution. To the first, no additional $\mathbf{6}_8$ solution was added, to the second 150 µL of $\mathbf{6}_8$ solution was added and to the last 300 µL of $\mathbf{6}_8$ solution was added. Each of these solutions was further divided into three solutions of 500 µL each. A magnetic cylindrical stirrer bar was added to each reaction vial and the solutions were kept at 30°C.

1.5 Reaction cycles involving chemical fuels

Sodium perborate solution (45 mM) used for oxidation was prepared by adding 6.92 mg of sodium perborate tetrahydrate in 1 mL of borate buffer. TCEP.HCl solution (45 mM) used for reduction was prepared by adding 12.89 mg of TCEP.HCl in 1.0 mL of borate buffer.

A solution of containing 90-95% $\mathbf{6}_8$ (520 µL, 0.45 mM in building block) was taken in three separate UPLC vials each sealed with screw caps. The solution was stirred at 1200 rpm and maintained at 30°C (see section SI 1.1 for details). TCEP solution was added to reduce the library around 10% and make it dynamic through generation of **6**.

Chemically fuelled reaction cycles were started by addition of TCEP (0.5, 0.6 or 0.7 eq.) solution. After 30 minutes of TCEP addition, sodium perborate, in an amount equivalent to TCEP, was added. After another 60 min of stirring, the first sample for UPLC measurement was prepared. The pH of the library throughout the experiment was 8-8.2.

1.6 Continuous flow experiment

A continuously stirred tank reactor (CSTR) operated under mass transport flow using syringe pumps was employed to conduct the experiment. Three syringe pumps (Chemyx Fusion 200), each equipped with one syringe (Hamilton glass 2500 µL, PTFE connector), were used. Two of these were used for infusion and one was used for withdrawal. A 2 mL vial, with a magnetic stirrer bar, maintained at 30°C and stirred at 1200 rpm was used as a CSTR. The vial was closed to arrest evaporation with a custom-built cap (Delrin) that contained three openings where three syringe tubings (PEEK) could be tightly screwed.

A solution of $\mathbf{6}_8$ (500 µL,0.45 mM in building block) was added to the CSTR. The solution was then reduced 10-15% using TCEP solution. The first inlet syringe was charged with a solution of **6** (0.9 mM) in buffer. In the second inlet syringe sodium perborate solution (0.9 mM) in buffer was placed. Both these solutions were degassed by passing nitrogen through them for 10 min prior to filling the syringes. The experiment was started by operating the infusion syringe pumps at 25 µL h^{-1} and the withdrawal syringe pump at 50 µL h^{-1} (turnover time of 600 minutes). After 3600 minutes (six turnover times), the syringe pumps were stopped.

1.7 Negative staining Transmission Electron Microscopy (TEM)

TEM samples were prepared by depositing a small aliquot (5 µL) of library solution onto a 400 mesh copper grid covered with a thin carbon film (van Loenen instruments). The grid was then blotted on filter paper. A solution of uranyl acetate (5 µL, 2%) was deposited on the grid and blotted with filter paper after incubation for 30 second. This was repeated once more to get appropriate staining. Transmission electron microscopy was performed on a Philips CM120 electron microscope operating at 120 kV. A slow scan CCD camera (Gatan) was used for recording images. ImageJ was used to analyse micrographs.

2. UPLC data

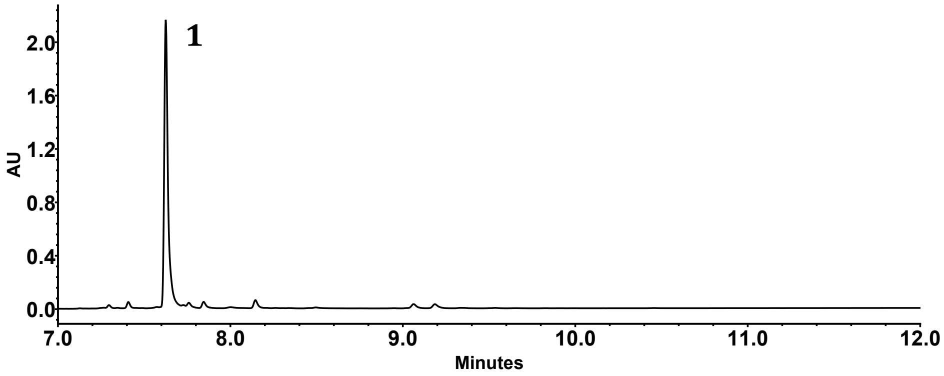

Figure S1. UPLC trace of the DCL made from **1** (0.45 mM) in borate buffer (50 mM, pH = 8.2) immediately after dissolving **1**.

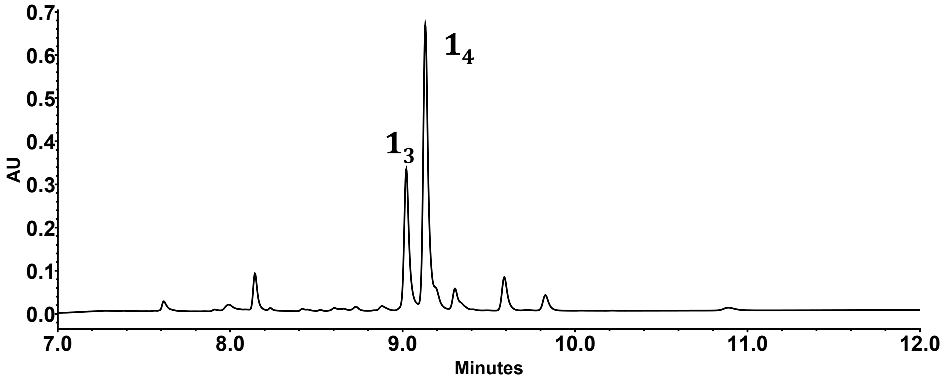

Figure S2. UPLC trace of the DCL made from **1** (0.45 mM) in borate buffer (50 mM, pH = 8.2) after stirring in presence of air for 22 days.

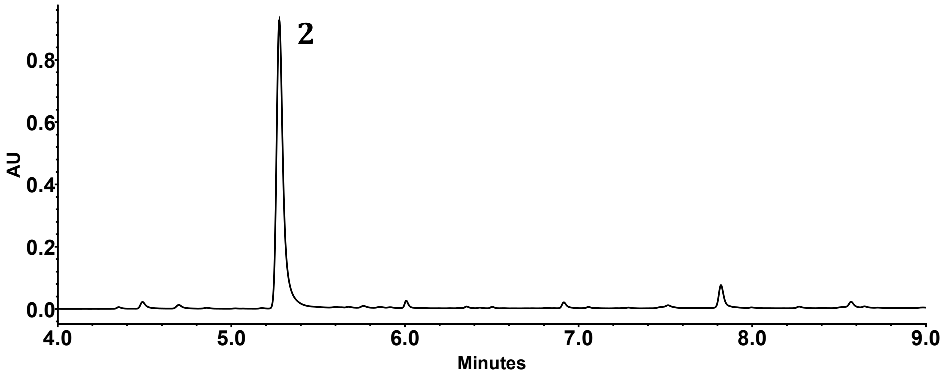

Figure S3. UPLC trace of the DCL made from **2** (0.45 mM) in borate buffer (50 mM, pH = 8.2) immediately after dissolving **2**.

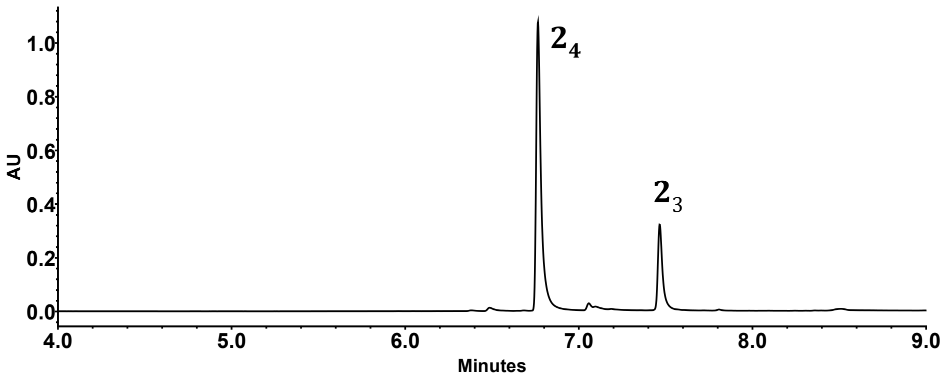

Figure S4. UPLC trace of the DCL made from **2** (0.45 mM) in borate buffer (50 mM, pH = 8.2) after stirring in presence of air for 22 days.

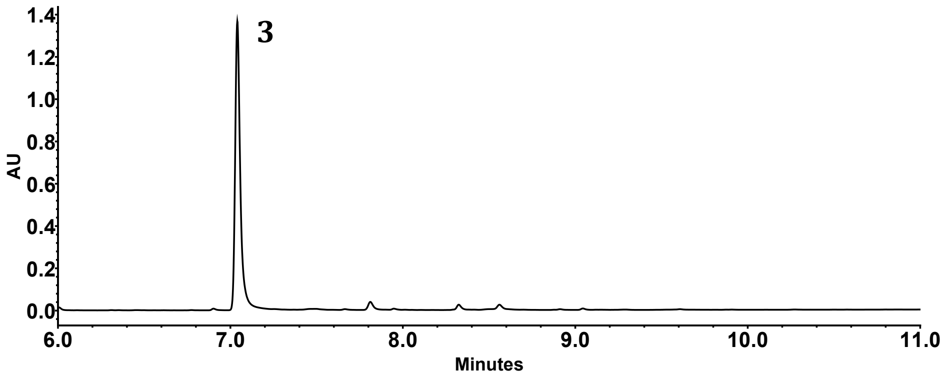

Figure S5. UPLC trace of the DCL made from **3** (0.45 mM) in borate buffer (50 mM, pH = 8.2) immediately after dissolving **3**.

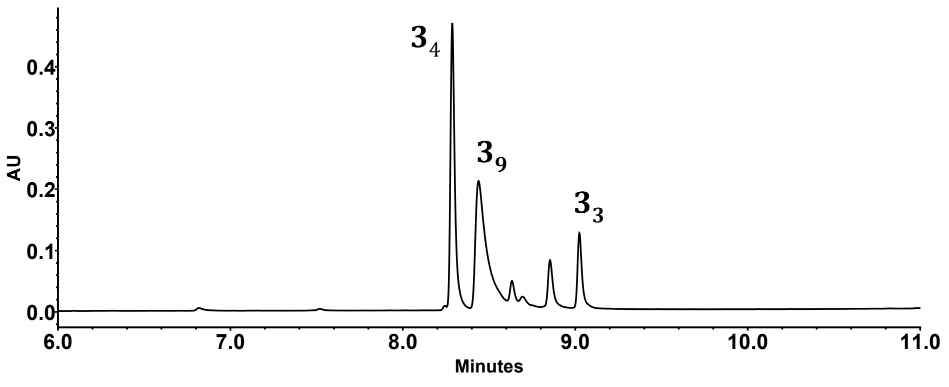

Figure S6. UPLC trace of the DCL made from **3** (0.45 mM) in borate buffer (50 mM, pH = 8.2) after stirring in presence of air for 22 days.

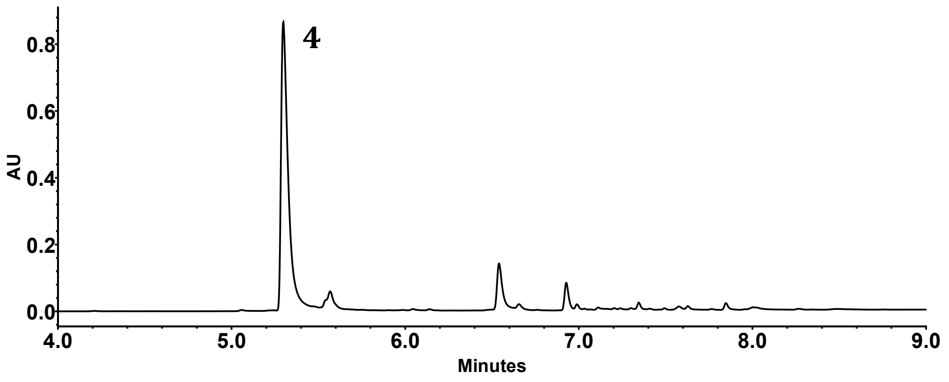

Figure S7. UPLC trace of the DCL made from **4** (0.45 mM) in borate buffer (50 mM, pH = 8.2) immediately after dissolving **4**.

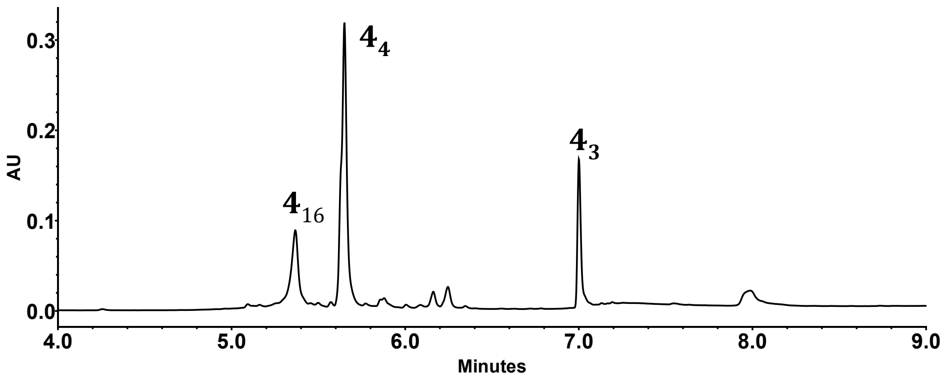

Figure S8. UPLC trace of the DCL made from **4** (0.45 mM) in borate buffer (50 mM, pH = 8.2) after stirring in presence of air for 22 days.

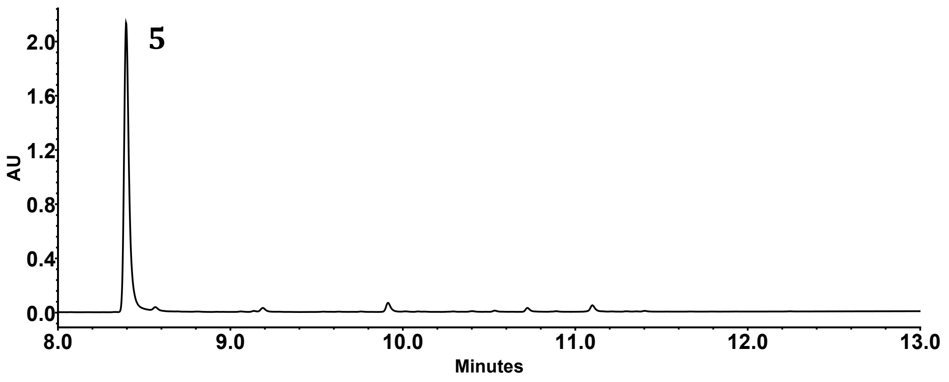

Figure S9. UPLC trace of the DCL made from **5** (0.45 mM) in borate buffer (50 mM, pH = 8.2) immediately after dissolving **5**.

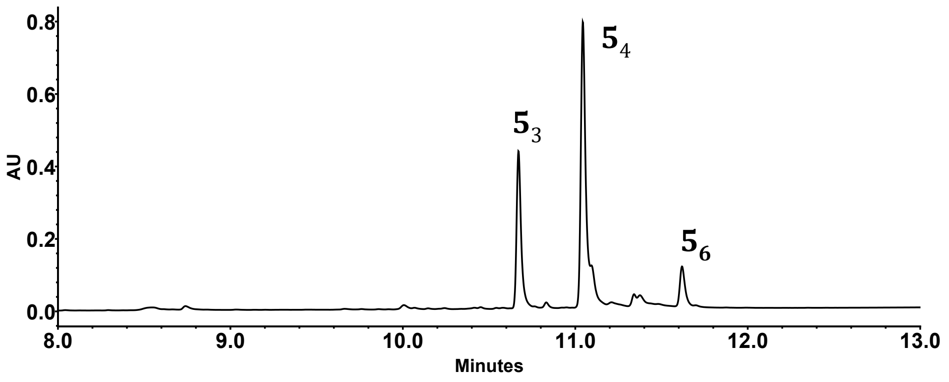

Figure S10. UPLC trace of the DCL made from **5** (0.45 mM) in borate buffer (50 mM, pH = 8.2) after stirring in presence of air for 22 days.

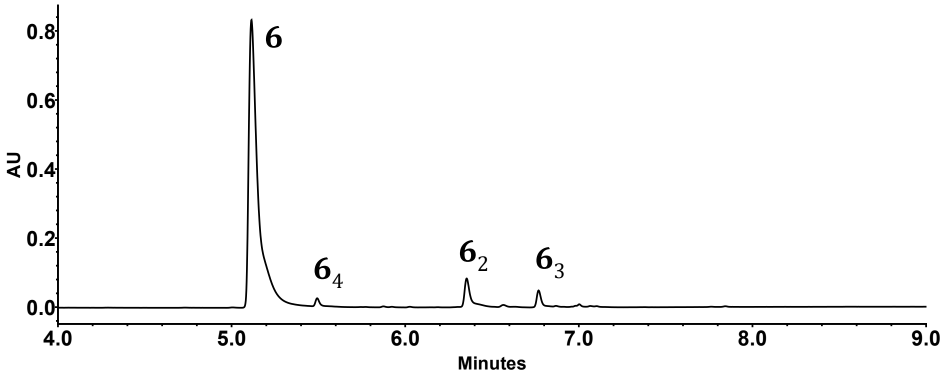

Figure S11. UPLC trace of the DCL made from **6** (0.45 mM) in borate buffer (50 mM, pH = 8.2) immediately after dissolving **6**.

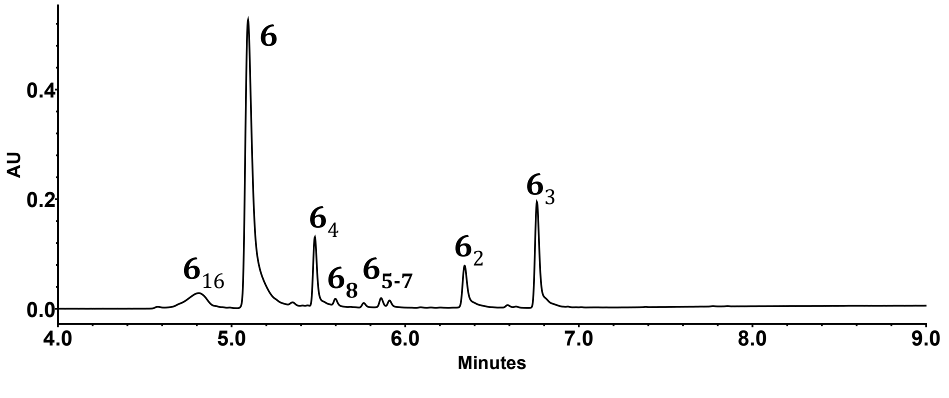

Figure S12. UPLC trace of the DCL made from **6** (0.45 mM) in borate buffer (50 mM, pH = 8.2) after stirring in presence of air for 56 hours.

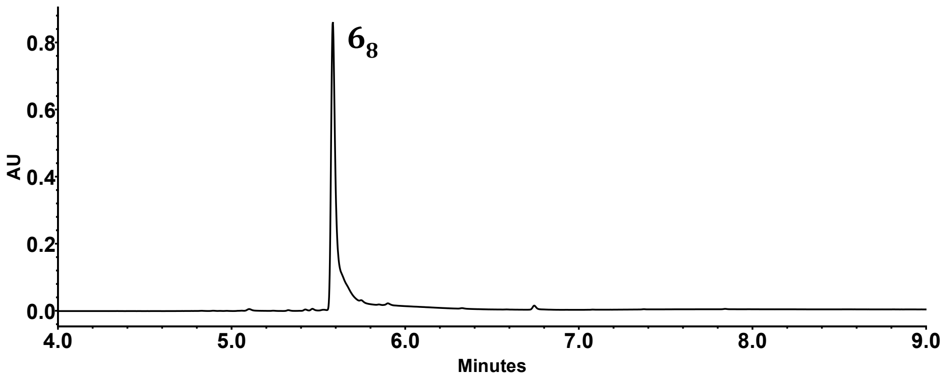

Figure S13. UPLC trace of the DCL made from **6** (0.45 mM) in borate buffer (50 mM, pH = 8.2) after stirring in presence of air for 242 hours.

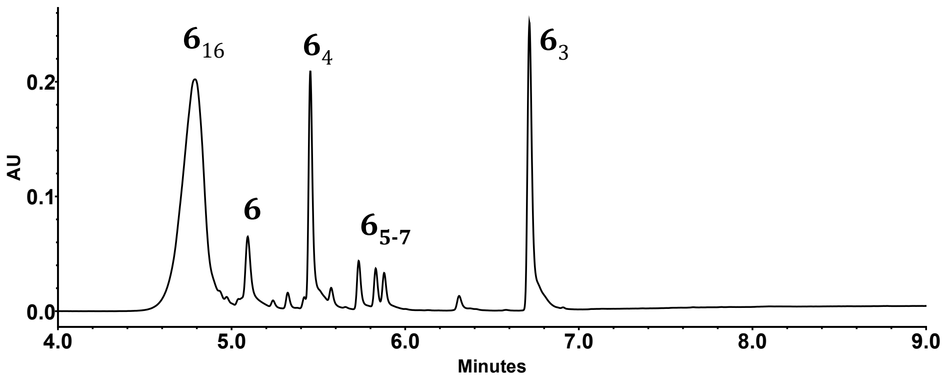

Figure S14. UPLC trace of the DCL made from **6** (0.45 mM) in borate buffer (50 mM, pH = 8.2) after standing in presence of air for 192 hours.

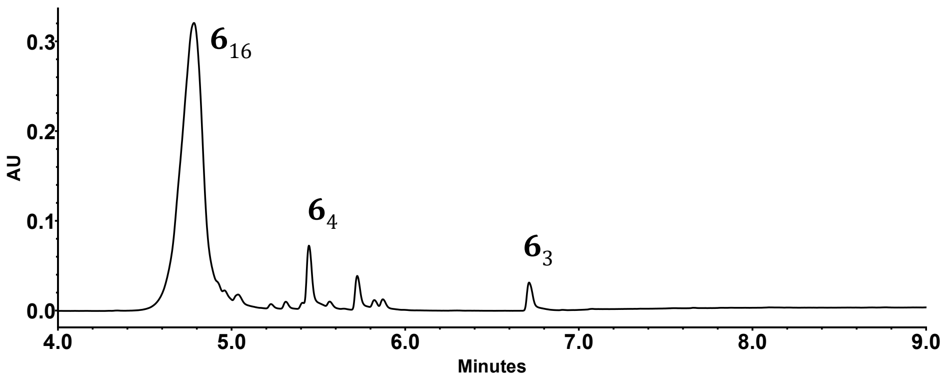

Figure S15. UPLC trace of the DCL made from **6** (0.45 mM) in borate buffer (50 mM, pH = 8.2) after standing in presence of air for 401 hours.

3. Peak identification with UPLC-MS

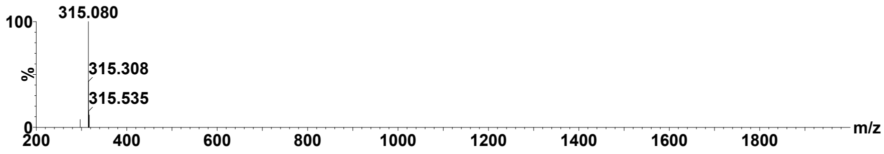

Figure S16 Mass spectrum of **6** from LC-MS analysis of the DCL made from **6** (corresponding to Fig S12). Calculated m/z: 315.082 $[M+H]^+$; observed m/z: 315.080 $[M+H]^+$.

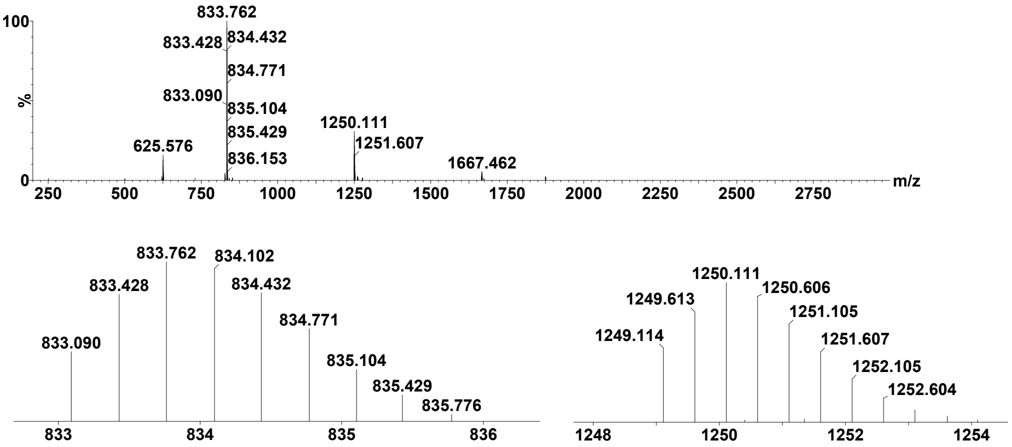

Figure S17. Mass spectrum of $\mathbf{6}_8$ from LC-MS analysis of the DCL made from **6** (corresponding to Fig S12). Calculated m/z: 833.495 $[M+3H]^{3+}$, 1249.742 $[M+2H]^{2+}$; observed m/z: 833.762 $[M+3H]^{3+}$, 1250.111 $[M+2H]^{2+}$.

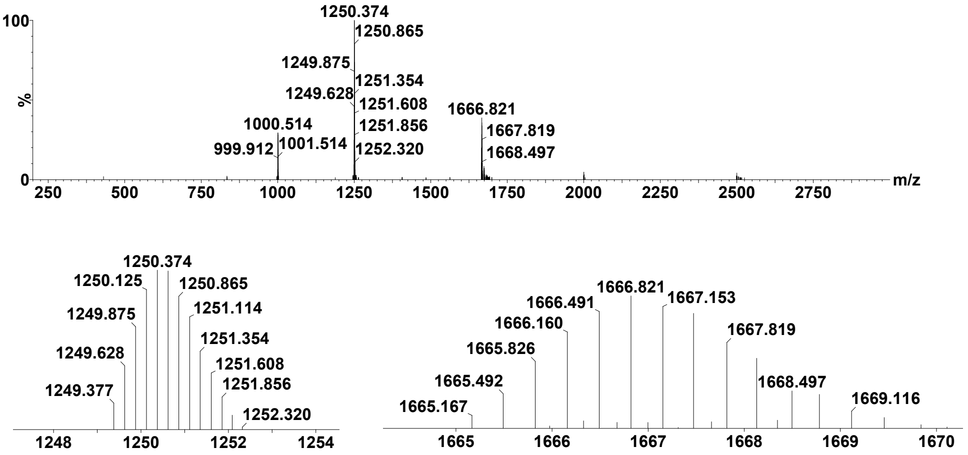

Figure S18. Mass spectrum of $\mathbf{6}_{16}$ from LC-MS analysis of the DCL made from **6** (corresponding to Fig S12). Calculated m/z: 1250.241 $[M+4H]^{4+}$, 1666.655 $[M+3H]^{3+}$; observed m/z: 1250.374 $[M+4H]^{4+}$, 1666.821 $[M+3H]^{3+}$.

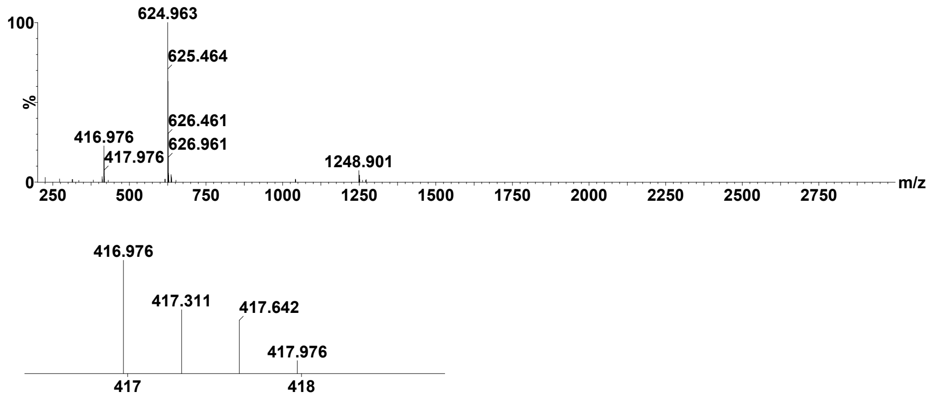

Figure S19. Mass spectrum of **6**4 from LC-MS analysis of the DCL made from f **6** (corresponding to Fig S12). Calculated m/z: 417.08 $[M+3H]^{3+}$, 625.12 $[M+2H]^{2+}$; observed m/z: 416.976 $[M+3H]^{3+}$, 624.963 $[M+2H]^{2+}$.

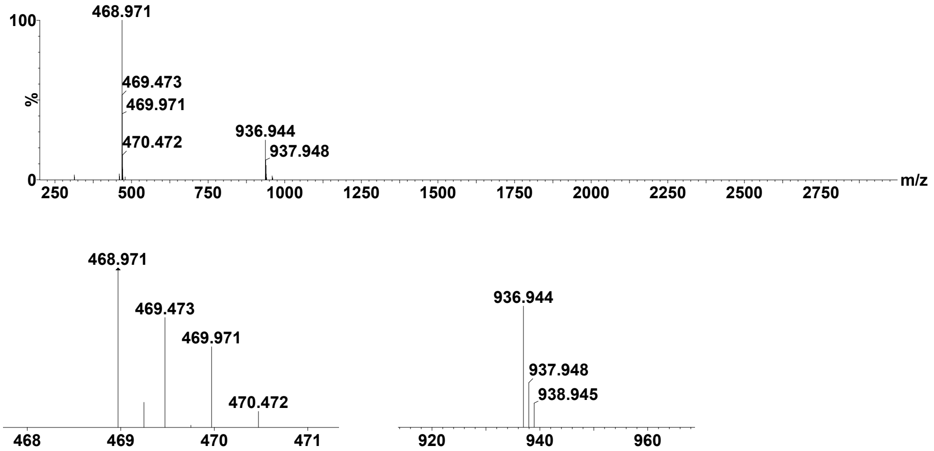

Figure S20. Mass spectrum of **6**3 from LC-MS analysis of the DCL made from **6** (corresponding to Fig S12). Calculated m/z: 469.09 $[M+2H]^{2+}$, 937.181 $[M+H]^{+}$; observed m/z: 468.971 $[M+2H]^{2+}$, 936.944 $[M+H]^{+}$.

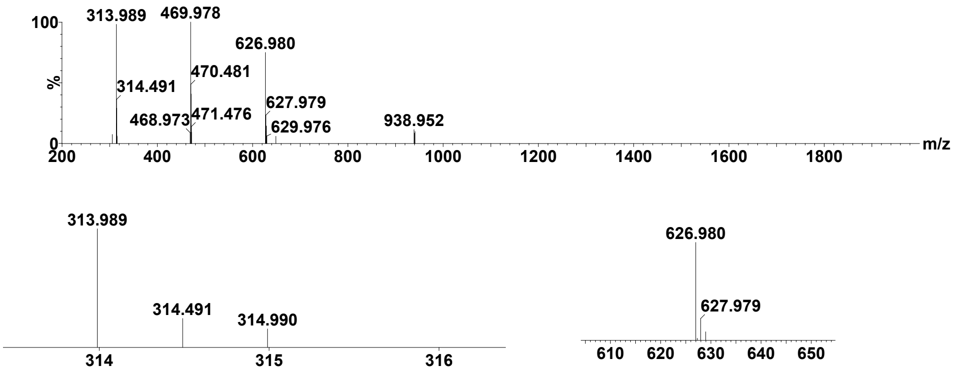

Figure S21. Mass spectrum of **6**2 from LC-MS analysis of the DCL made from **6** (corresponding to Fig S12). Calculated m/z: 314.068 $[M+2H]^{2+}$, 627.136. $[M+H]^{+}$; observed m/z: 313.989 $[M+2H]^{2+}$, 626.980 $[M+H]^{+}$. Peaks 469.978 $[M+2H]^{2+}$ and 938.952 $[M+H]^{+}$ correspond to **6**3.

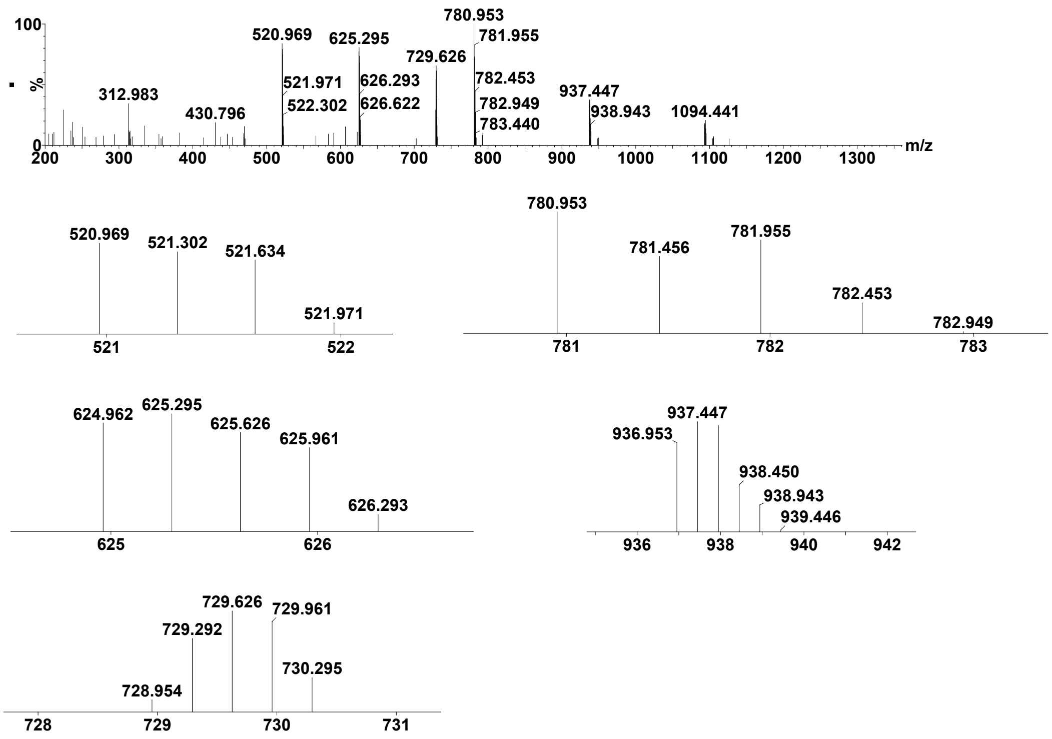

Figure S22. Mass spectrum of $\mathbf{6}_5$, $\mathbf{6}_6$ and $\mathbf{6}_7$ from LC-MS analysis of the DCL made from **6** (corresponding to Fig S12). $\mathbf{6}_5$: calculated m/z: 521.10 $[M+3H]^{+3}$, 781.15 $[M+2H]^{2+}$; observed m/z: 520.969 $[M+3H]^{3+}$, 780.953 $[M+2H]^{2+}$. $\mathbf{6}_6$: calculated m/z: 521.10 $[M+3H]^{+3}$, 937.18 $[M+2H]^{2+}$; observed m/z: 520.969 $[M+3H]^{3+}$, 937.447 $[M+2H]^{2+}$. $\mathbf{6}_7$: calculated m/z: 729.14 $[M+3H]^{+3}$; observed m/z: 729.626 $[M+3H]^{3+}$.

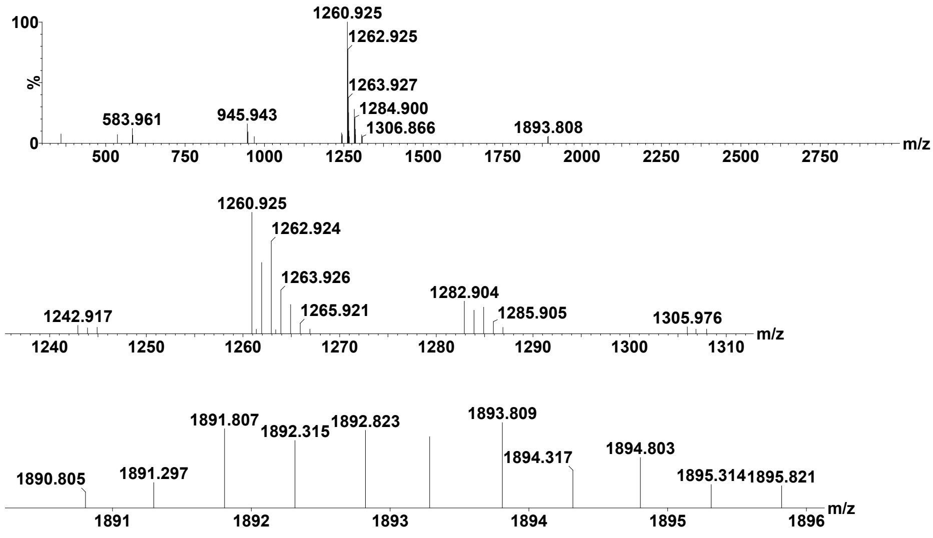

Figure S23. Mass spectrum of $\mathbf{1}_4$ from LC-MS analysis of the DCL made from **1** (corresponding to Fig S2). Calculated m/z: 1261.023$[M+H]^+$; observed m/z: 1260.925 $[M+H]^+$, m/z of 1282.904 corresponds to $[M+Na]^+$. Peaks at 1893.808 $[M+2H]^{2+}$ and 945.943 $[M+4H]^{4+}$ correspond three times the mass of $\mathbf{1}_4$.

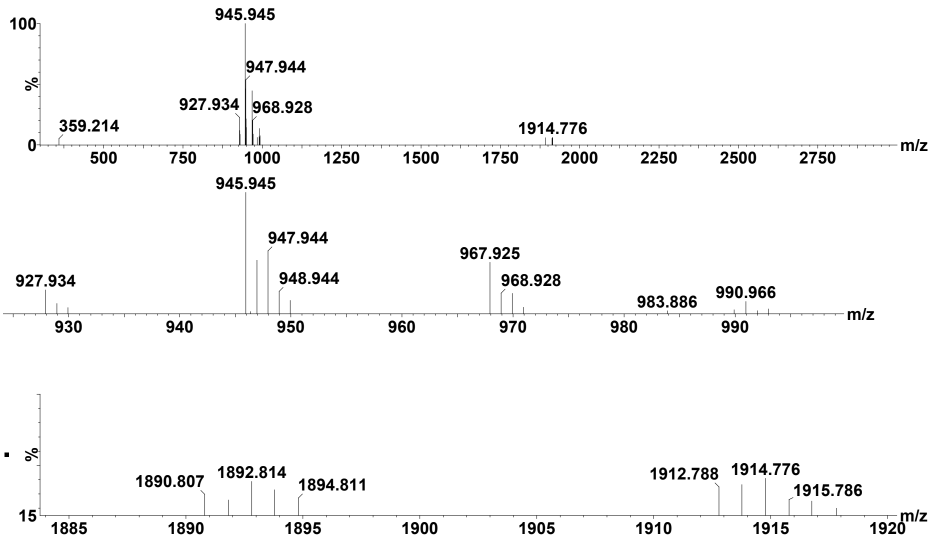

Figure S24. Mass spectrum of 1_3 from LC-MS analysis of the DCL made from **1** (corresponding to Fig S2). Calculated m/z: 946.017[M+H]$^+$; observed m/z: 945.945 [M+H]$^+$, m/z of 967.925 corresponds to [M+Na]$^+$. Peak at 1914.776 [M+Na]$^+$ corresponds to twice the mass of 1_3.

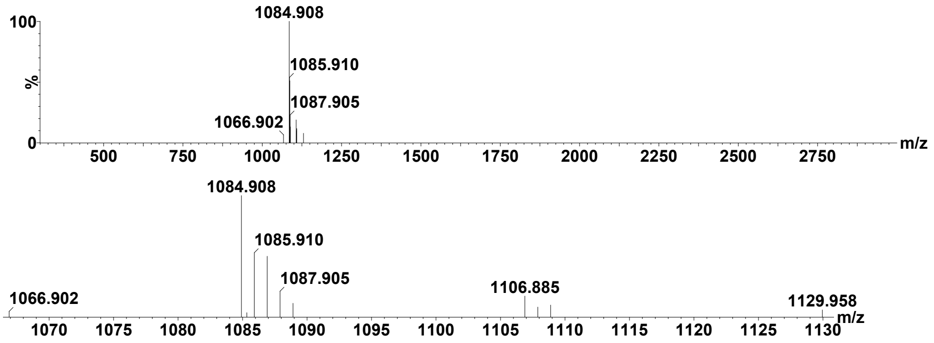

Figure S25. Mass spectrum of 2_4 from LC-MS analysis of the DCL made from **2** (corresponding to Fig S4). Calculated m/z: 1084.99 [M+H]$^+$; observed m/z: 1084.908 [M+H]$^+$. m/z of 1066.902 corresponds to [M-H_2O+H]$^+$, 1106.885 corresponds to [M+Na]$^+$.

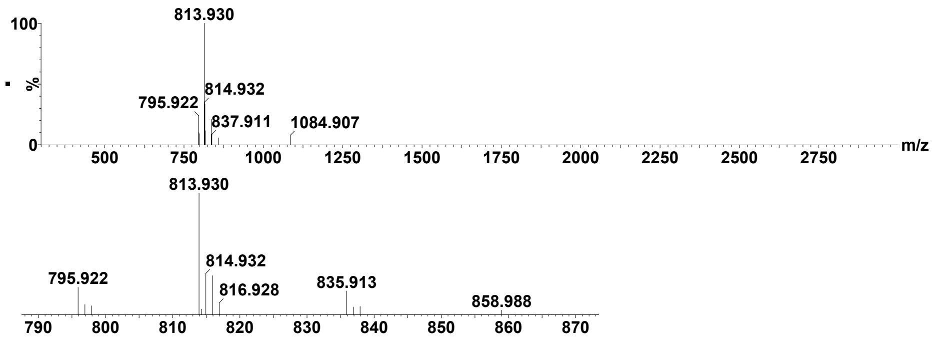

Figure S26. Mass spectrum of 2_3 from LC-MS analysis of the DCL made from **2** (corresponding to Fig S4). Calculated m/z: 813.99 [M+H]$^+$; observed m/z: 813.930 [M+H]$^+$. m/z of 795.922 corresponds to [M-H_2O+H]$^+$, 835.913 corresponds to [M+Na]$^+$.

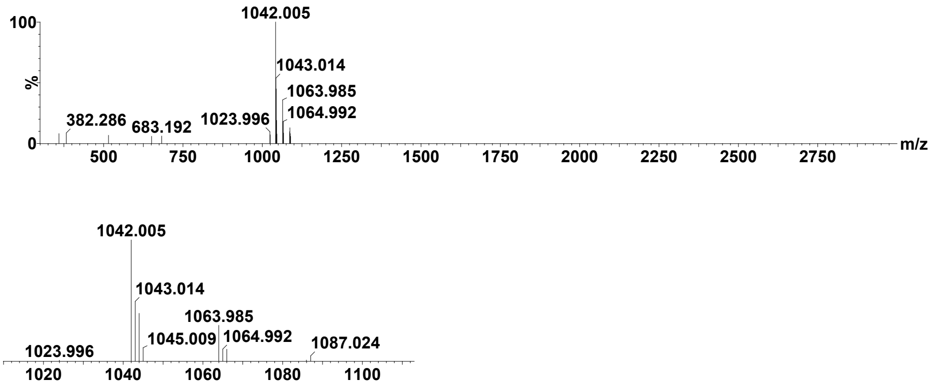

Figure S27. Mass spectrum of **3**$_3$ from LC-MS analysis of the DCL made from **3** (corresponding to Fig S6). Calculated m/z: 1042.086 [M+H]$^+$; observed m/z: 1042.005 [M+H]$^+$. m/z of 1023.996 corresponds to [M- H_2O +H]$^+$, 1063.985 corresponds to [M+Na]$^+$.

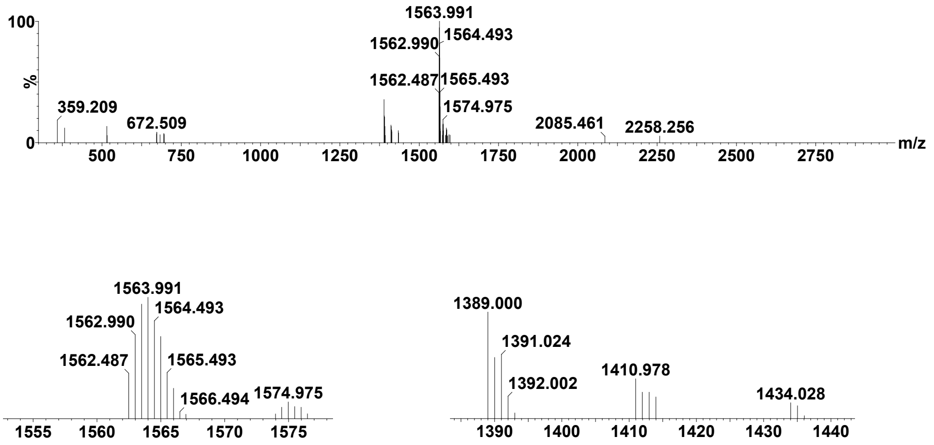

Figure S28. Mass spectrum of **3**$_9$ from LC-MS analysis of the DCL made from **3** (corresponding to Fig S6). Calculated m/z: 1563.130 [M+2H]$^{2+}$; observed m/z: 1563.991 [M+2H]$^{2+}$. m/z of 1389.000 [M+H]$^+$ corresponds to **3**$_4$.

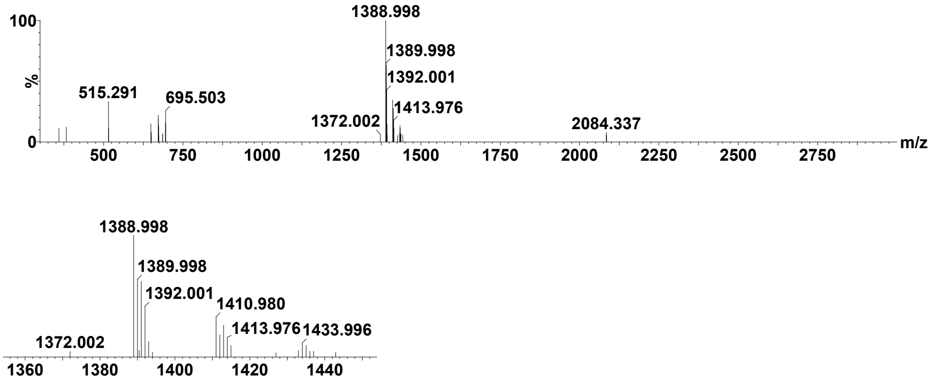

Figure S29. Mass spectrum of **3**$_4$ from LC-MS analysis of the DCL made from **3** (corresponding to Fig S6). Calculated m/z: 1389.114 [M+H]$^+$; observed m/z: 1388.998 [M+H]$^+$.

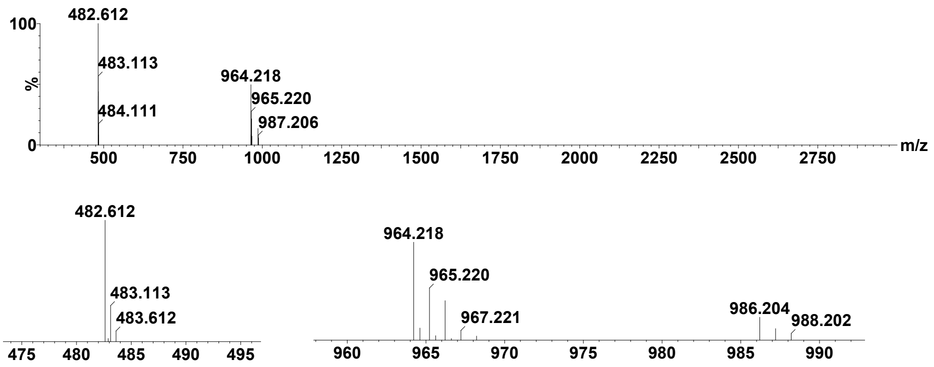

Figure S30. Mass spectrum of **4**$_3$ from LC-MS analysis of the DCL made from **4** (corresponding to Fig S8). Calculated m/z: 964.073 $[M+H]^+$, 482.536 $[M+2H]^{2+}$; observed m/z: 964.218 $[M+H]^+$, 482.612 $[M+2H]^{2+}$.

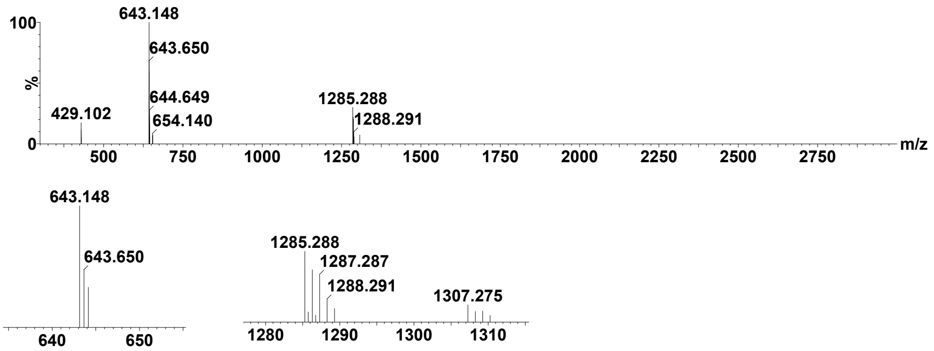

Figure S31. Mass spectrum of **4**$_4$ from LC-MS analysis of the DCL made from **4** (corresponding to Fig S8). Calculated m/z: 1285.097 $[M+H]^+$, 643.048 $[M+2H]^{2+}$, 429.032 $[M+3H]^{3+}$; observed m/z: 1285.288 $[M+H]^+$, 643.148 $[M+2H]^{2+}$, 429.102 $[M+3H]^{3+}$.

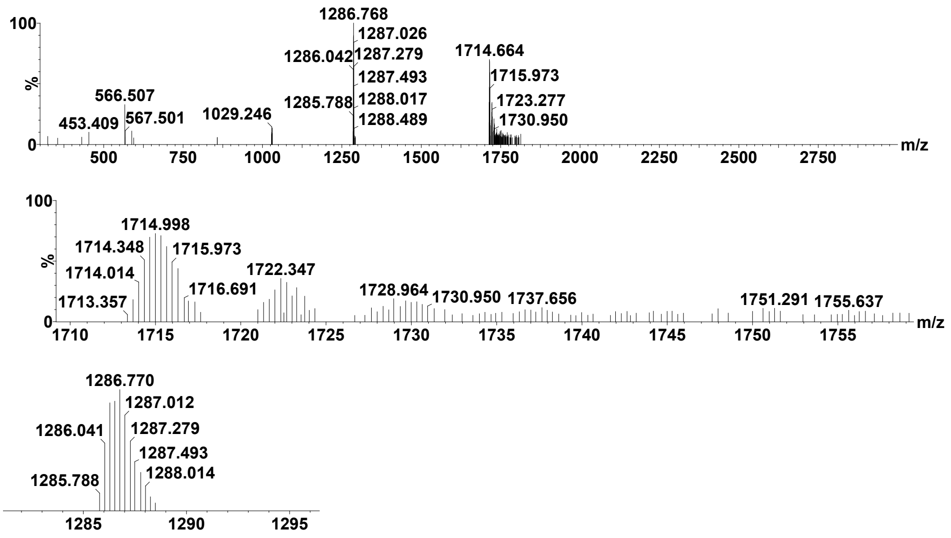

Figure S32. Mass spectrum of **4**$_{16}$ from LC-MS analysis of the DCL made from **4** (corresponding to Fig S8). Calculated m/z: 1719.807 $[M+3H]^{3+}$, 1290.105 $[M+4H]^{4+}$, 1032.284 $[M+5H]^{5+}$; observed m/z: 1714.664 $[M-H_2O+3H]^{3+}$, 1286.768 $[M- H_2O+4H]^{4+}$, 1029.246 $[M- H_2O +5H]^{5+}$.

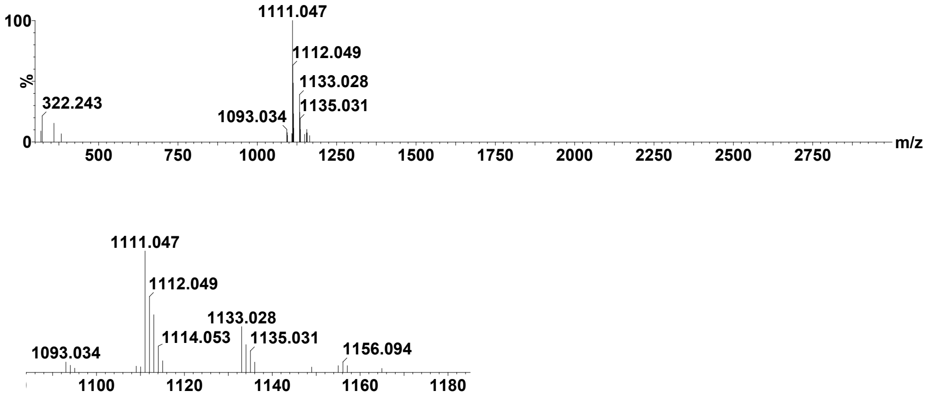

Figure S33. Mass spectrum of $\mathbf{5}_3$ from LC-MS analysis of the DCL made from **5** (corresponding to Fig S10). Calculated m/z: 1111.134 $[M+H]^+$; observed m/z: 1111.047 $[M+H]^+$, 1133.028 $[M+Na]^+$.

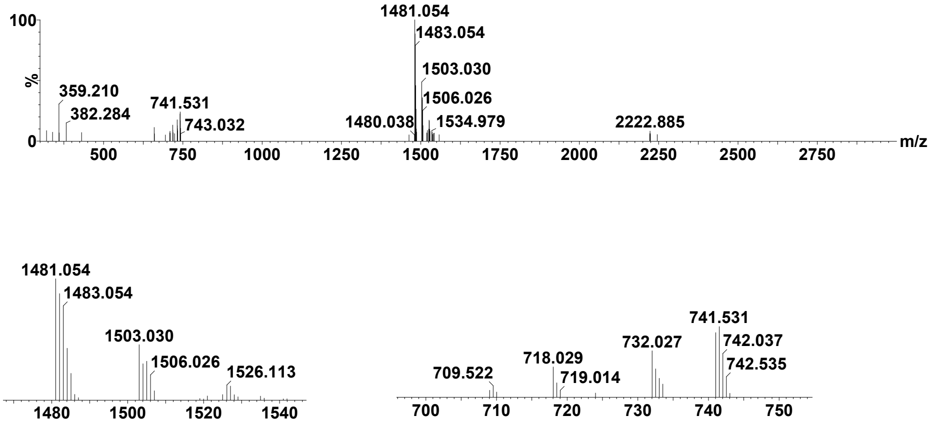

Figure S34. Mass spectrum of $\mathbf{5}_4$ from LC-MS analysis of the DCL made from **5** (corresponding to Fig S10). Calculated m/z: 1481.178 $[M+H]^+$, 741.089 $[M+2H]^{2+}$; observed m/z: 1481.054 $[M+H]^+$, 741.531 $[M+2H]^{2+}$.

4. Circular dichroism (CD) spectroscopy for $\mathbf{6}_8$ and $\mathbf{6}_{16}$

A JASCO J715 spectrophotometer and a HELMA quartz cuvette (1 mm path length) were used. Sample solutions were not diluted. The solvent spectrum was subtracted from all measurements. Spectra were recorded at 30°C between 190 and 500 nm at a scanning rate of 100 nm/min, 0.1 nm data pitch and 2 nm bandwidth. For each sample an average of three accumulations was used to obtain the spectrum. CD measurements were repeated over triplicates and plotted graphs show values averaged over those three measurements.

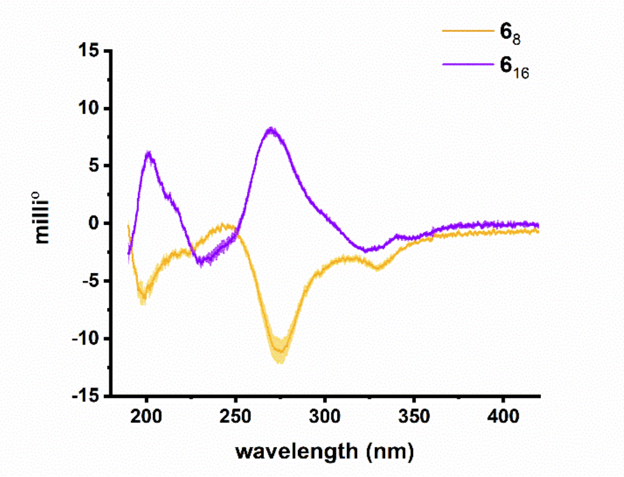

Figure S35. CD spectra of solutions (0.45 mM in building block) of **6**8 and **6**16 in borate buffer (50 mM) at 30°C.

5. Thioflavin T (ThT) fluorescence assay on 6₈ and 6₁₆

A JASCO FP6200 spectrophotometer and a HELMA quartz cuvette (10 mm path length) were used. Sample solutions were diluted to a concentration of 100 μM (in building block concentration) with additional borate buffer. The diluted samples (80 μL) were mixed with ThT solution (22 μM, 450 μL) in borate buffer, followed by incubation for ten minutes. An excitation wavelength of 440 nm (10 nm bandwidth) was used. Emission was recorded between 460 and 700 nm at scanning rate of 250 nm/min with three cycles. Spectra was recorded three times and the average values of these three measurements are reported with error.

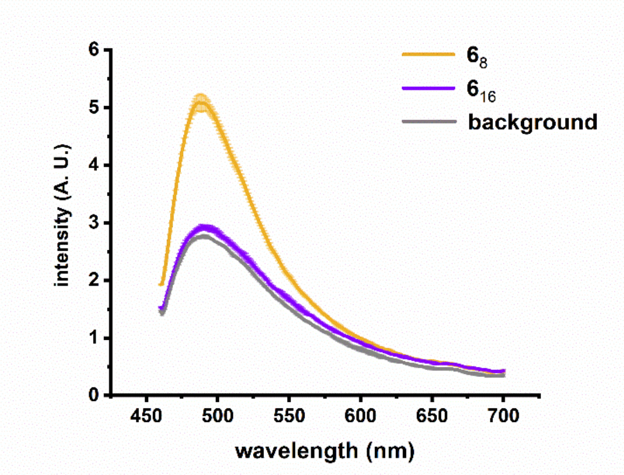

Figure S36. Thioflavin T fluorescence emission for **6**8 and **6**16 samples. Enhanced emission in **6**8 samples is indicative of binding sites that restrict the conformational freedom of the dye. Samples of **6**16 had nearly equal emission to the borate buffer (50 mM) suggesting absence of such binding sites.

6. MALDI profile of 6_{16}

Matrix solution was prepared by mixing acetonitrile (ULC-MS) and water (ULC-MS) in equal amounts with 0.1 V/V % of trifluoroacetic acid (ULC-MS) as a modifier. Library solution containing $\mathbf{6}_{16}$ was diluted to 0.1 mM (building block concentration) with matrix solution. A solution of α-cyano-4-hydroxycinnamic acid (CHCA) was prepared by dissolving 10 mg of CHCA in 1.0 mL of matrix solution. Three solutions were prepared by mixing diluted $\mathbf{6}_{16}$ solution with CHCA solution in 1:5, 1:10 and 1:20 volume ratio. These solutions were then spotted three times on a MALDI plate (384 Opti-TOF 123 x 81 mm; 1.0 µL per spot) and left to air dry for an hour. An applied Biosystems 4800 MALDI TOF/TOF spectrometer operated in linear positive mass mode was used to record spectra. Several mass ranges with 20 sub-spectra (containing 50 shots) each were analysed. Before every sub-spectrum the laser was moved to a different position of the spotted sample. mMass software was used for analysis of mass spectra. The fragmentation predominantly takes place at the disulfide bonds. The calculated m/z therefore corresponds to species containing n units of the building block without hydrogen atoms at the thiols.

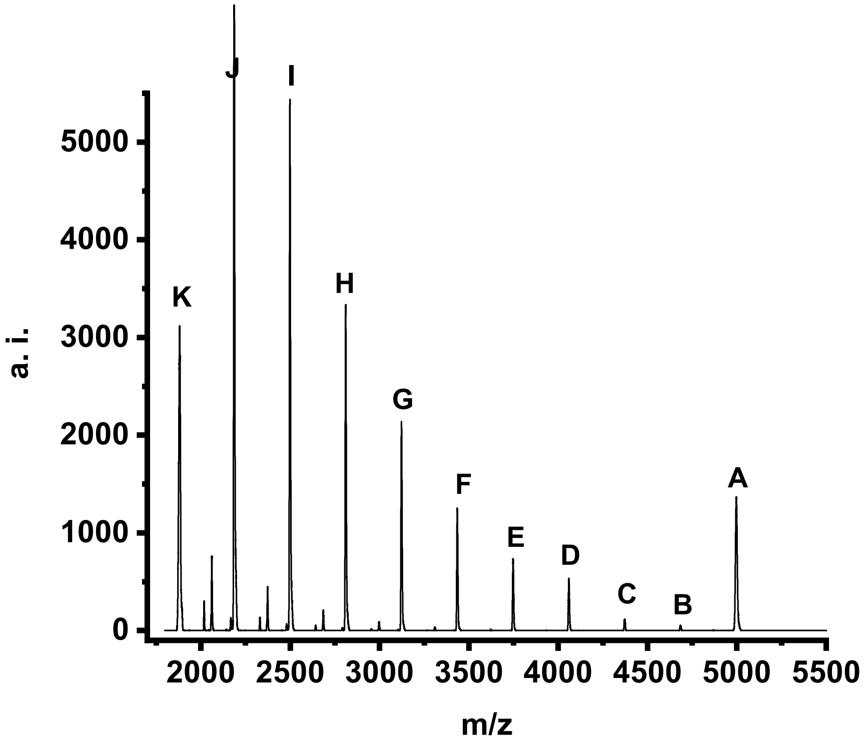

Figure S37. MALDI fragmentation pattern of $\mathbf{6}_{16}$. Fragmentation takes place predominantly at disulfide bonds.

Peak	Observed	Calculated (m/z)	Oligomer size
A	4996	4996.96	16
B	4684	4684.90	15
C	4372	4371.84	14
D	4059	4059.78	13
E	3747	3747.72	12

F	3435	3435.66	11
G	3122	3121.60	10
H	2810	2809.54	9
I	2498	2497.48	8
J	2186	2184.42	7
K	1881	1872.36	6

Table S1. Comparison of observed and calculated m/z from the MALDI fragmentation of $\mathbf{6}_{16}$ at the disulfide bonds. Presence of oligomers containing 16 to 8 units of building blocks confirms that $\mathbf{6}_{16}$ is a single macrocycle.

7. X-ray crystallography of $\mathbf{6}_{16}$

Aqueous solutions of **L-6**$_{16}$ and **D-6**$_{16}$ were prepared using pure water to a final concentration of 32 mg/ml. A racemic mixture was prepared by mixing the enantiopure solutions of **L-6**$_{16}$ and **D-6**$_{16}$. Crystallization screening was carried out using commercial random sparse matrix screen (JBScreen Wizard, Jena Bioscience) using the sitting drop vapour diffusion method, at 293 K. X-ray quality crystals (Figure S38) were optimized using the hanging drop method by mixing 1.2 µL of the racemic mixture **L/D-6**$_{16}$ and 1.8 µl of 20% v/v 1.4-butanediol, 100mM MES buffer (pH 6.0) and 200 mM lithium sulfate from the reservoir solution. A single crystal was fished using a MiTeGen microloop and plunged into liquid nitrogen directly such that the mother liquor served as cryoprotectant.

Synchrotron data was collected at P14 beamline operated by EMBL Hamburg at the Petra III storage ring (DESY, Hamburg, Germany) at a wavelength of 0.8 Å. During data collection the crystal was cooled to 100 K. The crystal was exposed for 0.008s and 0.1° oscillation per frame and a rotation pass of 360° was measured using an EIGER 16M detector. Diffraction data was processed using *CrysAlis*^{Pro}.[1] Though a racemic mixture was used for crystallization, the crystal contained one enantiomer only, the **D-6**$_{16}$ (Figure S39). The crystal belonged to space group $P3_221$ (or $P3_121$) with unit cell parameters $a = b = 29.065$ (2) Å, $c = 41.004$ (3) Å, $V = 29999.2$ Å^3 and ½ molecule in the asymmetric unit ($Z = 3$). The structure was solved with *SHELXT*[2] structure solution program using Intrinsic Phasing. The structure was refined by the full-matrix least-squares method on F2 with *SHELXL-2014*[3] within *Olex2*.[4] After each refinement step, visual inspections of the model and electron density maps were carried out using *Olex2*[4] and *Coot*[5]. Most of the side chain atoms of lysines were observed to be severely disordered and were either omitted or refined with partial occupancy. AFIX, DFIX, SADI and FLAT instructions were used to improve the geometry of the molecules. Restraints on anisotropic displacement parameters were implemented with DELU, SIMU and EADP instructions. After several attempts to model the disordered side chains, the SQUEEZE[6] procedure was used to flatten the electron density map. The calculated total potential solvent accessible void volume and electron counts per cell were 17957.5 Å^3 and 2461 respectively. Hydrogen atoms were placed at idealized positions except for those at disordered or missing side chains.

Statistics of data collection and refinement of **6**$_{16}$ are described in Table S2. The final CIF file of **6**$_{16}$ was examined in IUCr's checkCIF algorithm. Due to the large volume fractions of disordered solvent molecules, weak diffraction intensity and poor resolution, a number of A- and B- level alerts remain in the checkCIF file. These alerts are inherent to the data set and refinement procedures. They are listed below and were divided into two groups. The first group demonstrates weak quality of the data and refinement statistics when compared to those expected for small molecule structures from highly diffracting crystals. The second group is related to decisions made during refinement and explained below. Atomic coordinates and structure factors of **6**$_{16}$ were deposited in the Cambridge Crystallographic Data Centre (CCDC) with accession code 2210295. The data is available free of charge upon request (www.ccdc.cam.ac.uk/).

CheckCIF validation of 6$_{16}$**:**

Group 1 alerts (these illustrate weak quality of data and refinement statistics if compared to small molecule structures from highly diffracting crystals):

THETM01_ALERT_3_A The value of sine(theta_max)/wavelength is less than 0.550
Calculated sin(theta_max)/wavelength = 0.5000
PLAT082_ALERT_2_A High R1 Value .... 0.21 Report
PLAT084_ALERT_3_A High wR2 Value (i.e. > 0.25) .... 0.50 Report
PLAT230_ALERT_2_B Hirshfeld Test Diff for S1 --C53 ....10.1 s.u.
PLAT234_ALERT_4_A Large Hirshfeld Difference .... 0.36 Ang.
PLAT414_ALERT_2_A Short Intra D-H..H-X ....1.54 Ang.
PLAT430_ALERT_2_A Short Inter D...A Contact O2AA ..O34 . ...2.45 Ang.
PLAT241_ALERT_2_B High/Low 'MainMol' Ueq as Compared ...... Check
PLAT315_ALERT_2_B Singly Bonded Carbon Detected (H-atoms Missing). C30 Check
PLAT340_ALERT_3_B Low Bond Precision on C-C Bonds ......0.03019 Ang.

Group 2 alert (is connected with decision made during refinement and explained below):

PLAT201_ALERT_2_B Isotropic non-H Atoms in Main Residue(s) 3 Report

C30 C1AA C61

These belong to the disordered Lysine sidechains that were refined with isotropic displacement parameters.

Identification code	D-**6**$_{16}$
Empirical formula	$C_{168} H_{160} N_{18} O_{30} S_{32}$
Formula weight	3937.5
Temperature	100 K
Wavelength	0.800 Å
Crystal system	Hexagonal
Space group	$P3_221$

Unit cell dimensions	*a* = *b* = 29.065 (2) Å ,*c* = 41.004 (2) Å
Volume	29999.2 (5) Å^3
Z	3
Density (calculated)	0.654 g/cm^3
Absorption coefficient	0.280 μ/mm^{-1}
Colour and shape	Colorless, blocks
Crystal size	0.120 x 0.100 x 0.050 mm
Index ranges	-29 ≤ h ≤ 29<br>-29 ≤ k ≤ 29<br>-41 ≤ l ≤ 41
Reflections collected	176167
R_{int}	0.1155
Data/restraints/parameters	20867/276/468
Goodness-of-fit on F^2	2.017
Final R indexes [$I > 2\sigma(I)$]	R_1 = 0.2054<br>wR_2 = 0.4574
Final R indexes [all data]	R_1 = 0.2213<br>wR_2 = 0.5007
Largest diff. peak and hole	1.23/-0.52 e Å^{-3}
Total potential solvent accessible void volume from SQUEEZE	17957.5 Å^3
Electron count/cell	2461
CCDC #	2210295

Table S2. Crystallographic data and refinement details for **6**$_{16}$.

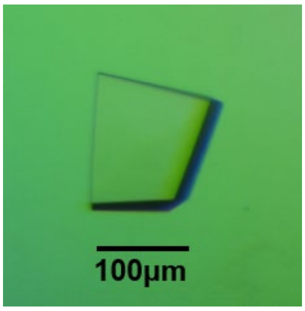

Figure S38. Single crystal of **6**$_{16}$ observed under cross-polarizing microscope.

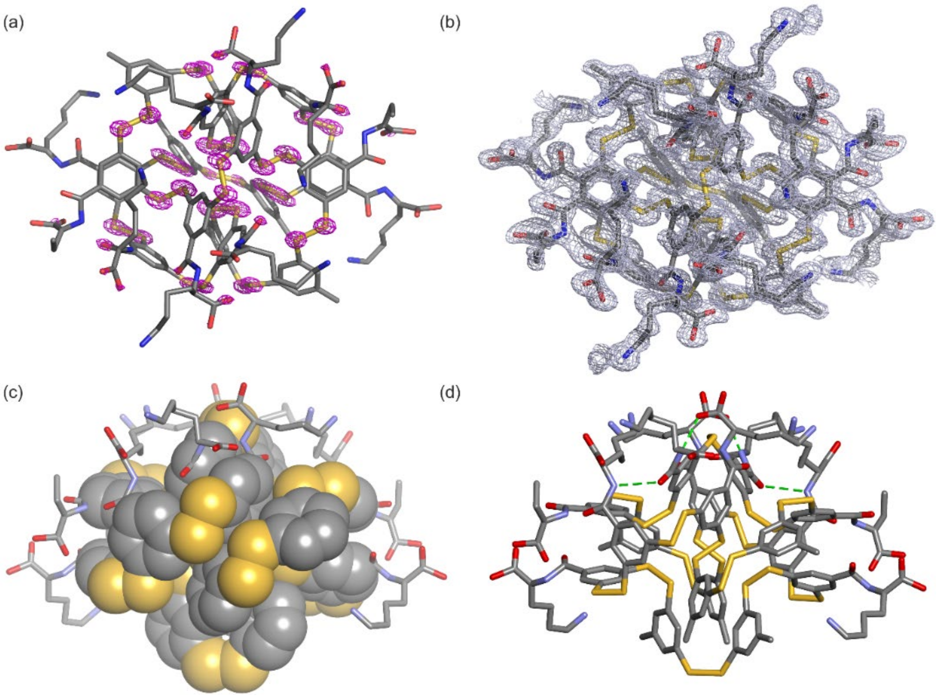

Figure S39. Crystal structure of the **D-6**$_{16}$ foldamer. (a,b) Sigma weighted 2F_o-F_c electron density maps superimposed on **D-6**$_{16}$ macrocycle. (a) Magenta mesh, contoured at 4 σ level shows the position of sulfur atoms and (b) grey mesh, contoured at 1 σ level shows the shape of macrocycle. (c) Space filling representation of the hydrophobic dimercaptobenzene core in grey (benzene rings) and gold (disulfide bonds). Side chains are shown in tubes. Hydrogen atoms omitted for clarity. (d) Hydrogen bonds that involve peptide sidechains are shown as green dashed lines.

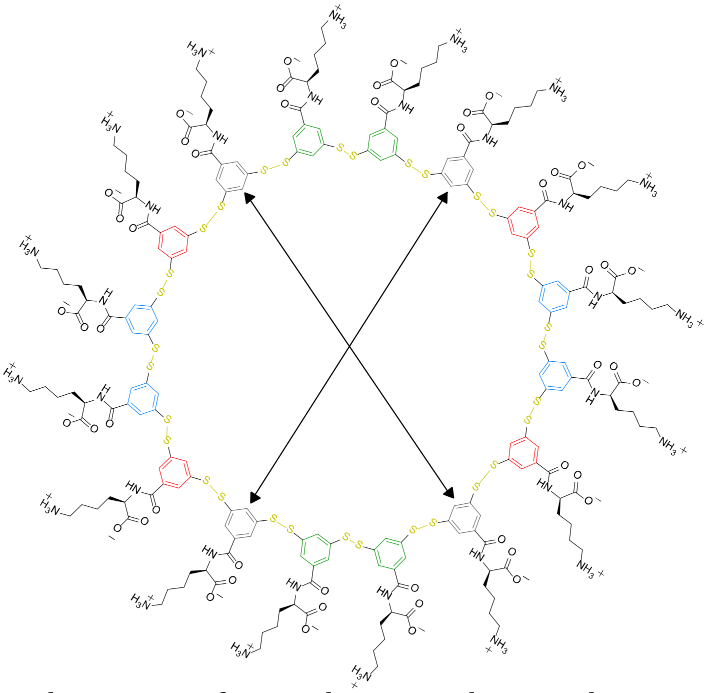

Figure S40. Chemical structure of **6**$_{16}$ with arrows showing the pairs of phenyl rings (grey) that are involved in π-stacking in the X-ray crystal structure of D-**6**$_{16}$. Color coding is used to denote phenyl rings that are identical by symmetry.

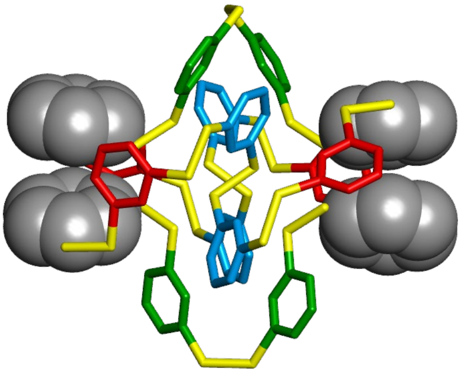

Figure S41. X-ray crystal structure of D-**6**$_{16}$ showing the phenyl rings involved in π-stacking in CPK representation. Color coding is used to denote phenyl rings that are identical by symmetry and correspond to Figure S40.

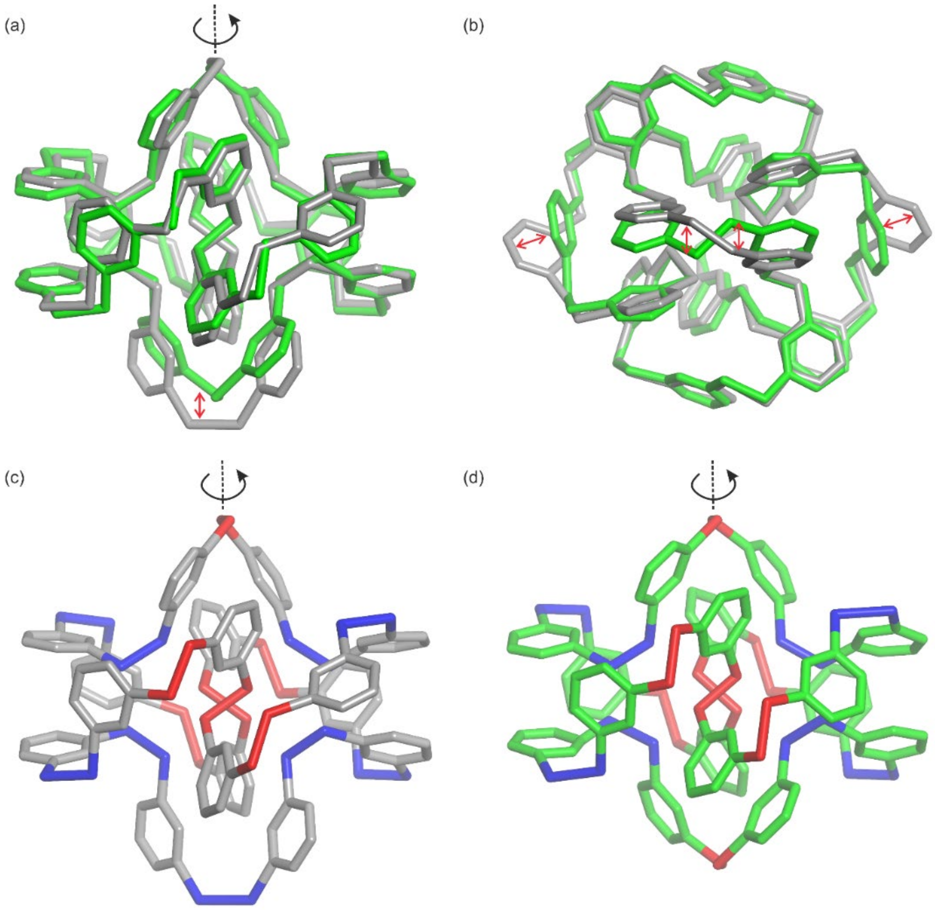

Figure S42. (a,b) Different views of tube representation of the hydrophobic core of D-**6**$_{16}$ (grey) aligned sterically to the hydrophobic core of an analogous hexadecamer structure (green) formed from the assembly of sixteen units of a building block bearing dipeptide sidechains Phe(4-guanidium) and Lys .[7] Note that the chirality of the analogous hexadecamer structure has been inverted with respect to the original publication to facilitate the comparison with the structure of D-**6**$_{16}$. Red arrows in (b) shows the deviations of the two hexadecamer hydrophobic cores. (c,d) Benzene rings of D-**6**$_{16}$ and analogous hexadecamer shown in grey and green, respectively, and the disulfide bonds in red or blue depending on their *M* or *P* chirality, respectively, clearly showing that the two hexadecamer hydrophobic cores are different.

8. DOSY NMR spectra of 6_{16}

A Bruker AVANCE NEO 600 spectrometer was used to acquire the diffusion-ordered NMR spectra of **6**$_{16}$ at 600 MHz frequency and 298K temperature with Bruker pulse program stebpgp1s. Diffusion time was adjusted to 150 ms. Using a linear ramp, the pulse gradient was increased. The value was increased from 2% of the maximum gradient strength to 95%. A total of 16 data points were collected for F1 dimension and 65,000 data points (12 ppm) were collected for F2 dimension.

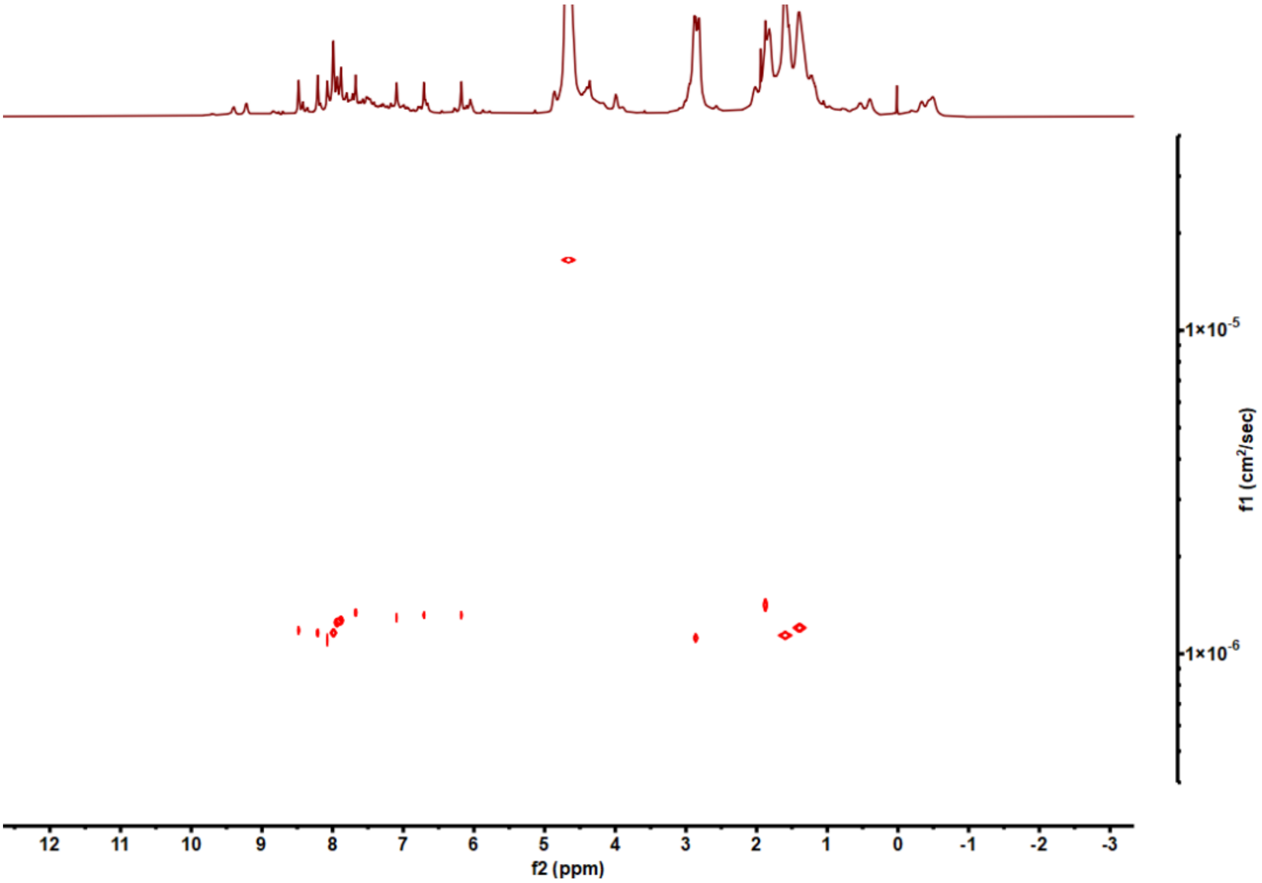

Figure S43. DOSY NMR spectrum of **6**$_{16}$ at 298 K.

9. Variable temperature response – CD and UPLC - of 6_{16} in solution

9.1 CD

A Peltier temperature controller was used to vary the temperature starting from 5 °C and ending at 65 °C in steps of 5 °C/min. Temperature was maintained for 10 min at every value before taking the measurement. The sample was allowed to cool to 30 °C over 65 hours and a spectrum was recorded again. Spectra were recorded between 190 and 500 nm at a scanning rate of 100 nm/min, 0.1 nm data pitch and 2 nm bandwidth. For each sample the average of three accumulations was used to obtain the spectrum. CD measurements were repeated in triplicate and plotted graphs show values averaged over those three measurements.

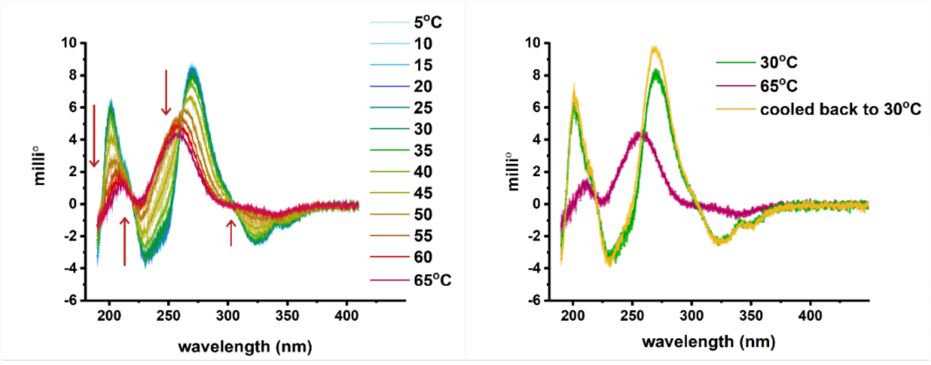

Figure S44. CD signal from **6**16 in solution reduces as temperature is increased (left). CD signal is recovered after the solution is left to cool at 30°C for 65 hours.

9.2 UPLC

A 500 µL solution of **6**16 (0.45 mM, pH = 8) was taken in a 2 mL vial. The vial was placed in a custom milled aluminium block in a Thermo Fischer compact dry bath and the solution was stirred at 1200 rpm. The temperature was raised to 35 °C and kept constant for 20 min. The vial was then placed inside the UPLC sample manager maintained at the same temperature as the dry bath. The library solution (10 µL) was injected directly. The vial was then placed back into the dry bath. The process was repeated with increments of 10 °C till 65 °C. The solution was then allowed to cool and two more measurements were taken. First, after the solution had cooled to 30 °C in an hour and, second, after allowing the solution to stand at room temperature for 65 hours.

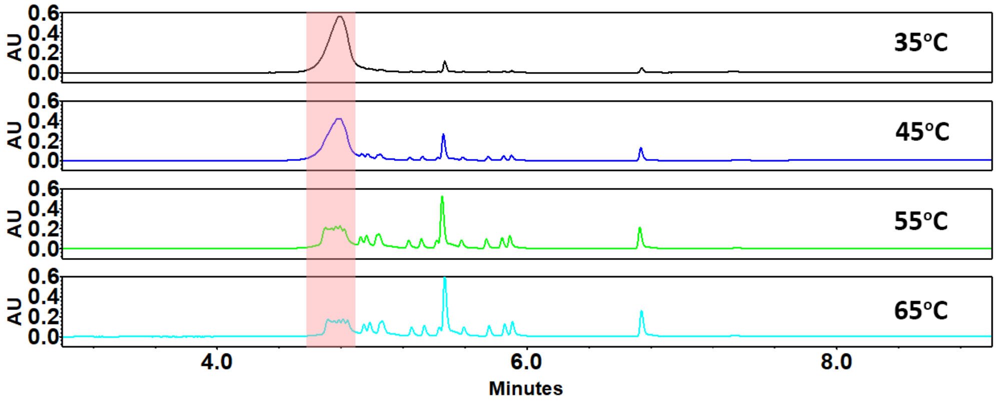

Figure S45. UPLC chromatograms of the **6**16 library after heating at different temperatures for 20 minutes followed immediately by UPLC analysis. At 55 °C, several peaks are seen at same retention time as **6**16 along with smaller macrocycles.

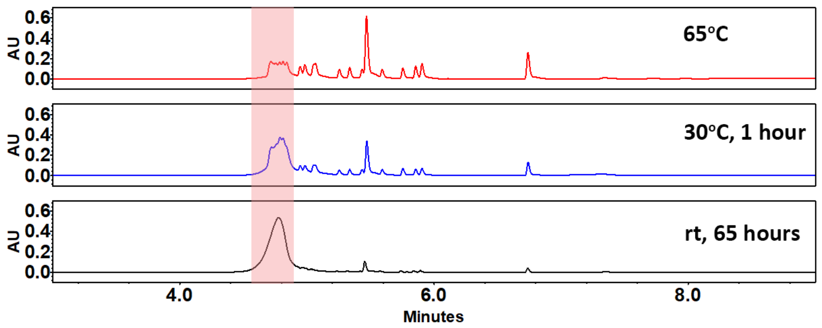

Figure S46. UPLC chromatogram of the **6**16 library immediately after heating for 20 min at 65 °C (top), one hour after cooling to 30 °C (middle) and 65 hours after maintaining at room temperature.

Analysis – Upon heating, the **6**16 peak in UPLC chromatogram split into several peaks with similar retention times (4.68 to 4.90, highlighted in pink). The splitting decreased when sample was allowed to cool. All the peaks showed the same mass as **6**16. We interpret these peaks as observation of partially unfolded states of **6**16 brought about by heating. Heating was also accompanied with an increase in the amounts of smaller macrocycles. This observation can mean that unfolding of **6**16 and loss of stabilising interactions of the fold result in its conversion to smaller macrocycles. Alternatively, it is possible that higher temperature shifts the equilibrium to smaller macrocycles. The reduction in CD signal hints at the loss of compact fold resulting from heating.

10. TCEP reduction of a mixture of 68 and 616

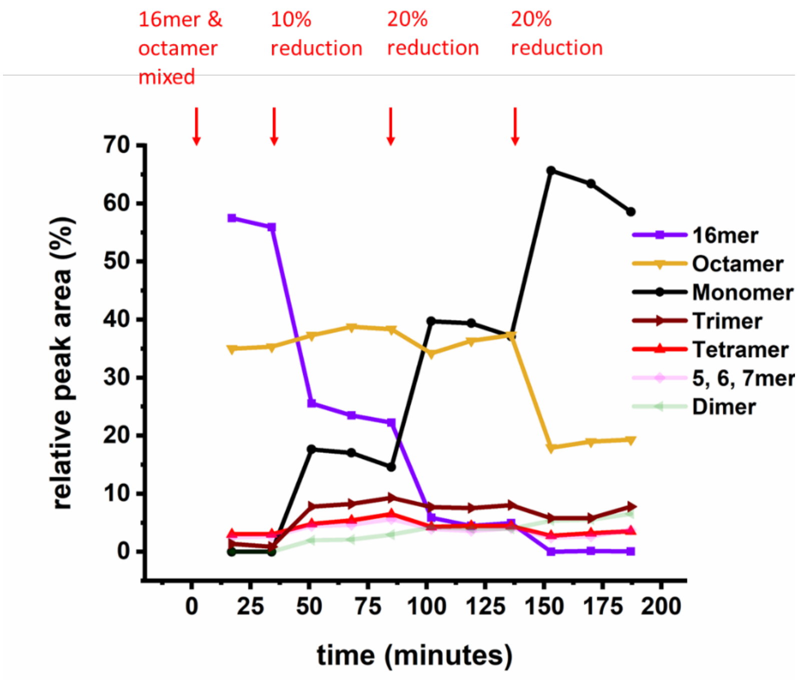

Figure S47. Change in composition of DCL of **6** monitored by UPLC (absorbance at 254 nm) as a roughly equimolar solution in **6**8 and **6**16 is reduced by successive additions of TCEP solution. Lines are drawn to guide the eye.

11. References

1. CrysAlisPRO, Oxford Diffraction/Agilent Technologies Ltd, Yarnton, England.
2. G. M. Sheldrick, *Acta Crystallogr., Sect. A,* **2015**, *71*, 3–8.
3. G. M. Sheldrick, *Acta Crystallogr., Sect. C,* **2015**, *71*, 3–8.
4. O.V. Dolomanov, et al., *J. Appl. Cryst.* **2009**, *42*, 339–341.
5. P. Emsley, et al., *Acta Crystallogr., Sect. D*, **2010**, *66*, 486–501.
6. A. L. Spek, *Acta Crystallogr., Sect. D*, **2009**, *65*, 148–155.
7. Pappas, C. G., et al., *Nat Chem.* **2020**, *12* , 1180-1186.
